# Microbial Community Dynamics in Natural *Drosophila melanogaster* Populations Across Seasons

**DOI:** 10.1111/1462-2920.70104

**Published:** 2025-06-02

**Authors:** Marion Margaux Lemoine, Thomas Wöhner, Martin Kaltenpoth

**Affiliations:** ^1^ Department of Insect Symbiosis Max Planck Institute for Chemical Ecology Jena Germany; ^2^ Julius Kühn‐Institut (JKI), Federal Research Centre for Cultivated Plants Institute for Breeding Research on Fruit Crops Dresden Germany

**Keywords:** bacterial community, *Drosophila melanogaster*, fungal community, indicator species, insect‐host interactions, seasonal adaptation, symbiosis

## Abstract

Many insects benefit from gut microbes that contribute to digestion, detoxification, nutrient supplementation or defence. Although abiotic and biotic factors are known to shape insect‐associated microbial communities, the seasonal dynamics and their potential impact on host fitness remain poorly studied. Here we investigated the temporal changes in bacterial and fungal communities associated with the model organism *Drosophila melanogaster* over 5 months. Our results reveal high inter‐individual variation, but also consistent changes in microbial communities of three wild *D. melanogaster* populations from early spring to late summer. These changes were driven by specific indicator species, particularly Acetobacteraceae bacteria (*Gluconobacter* and *Komagataeibacter*) and Saccharomycetales yeasts (*Pichia*, *Starmerella*, *Kregervanrija*, *Hanseniaspora*, *Saccharomycopsis*, *Priceomyces* and *Dipodascopsis*). The temporal dynamics were not accompanied by differences in the total bacterial or fungal abundance, and alpha‐diversity only changed across sampling months for the fungal but not the bacterial communities. While the changes in *D. melanogaster* ‐associated microbial communities are likely driven by the exposure to seasonally changing microbial environments and diets, they may have important impacts on host fitness. Elucidating the potential adaptive value of seasonally changing microbial communities will enhance our understanding of how symbiotic microbes may contribute to ecological niche shifts and geographic range expansions in insects.

## Graphical abstract

The characterisation of fungal and bacterial communities associated with three field populations of *Drosophila melanogaster* in Germany revealed consistent but also population‐specific changes across seasons. The seasonal microbiota variation was mostly driven by indicator species of Acetobacteraceae bacteria and Saccharomycetales yeasts.

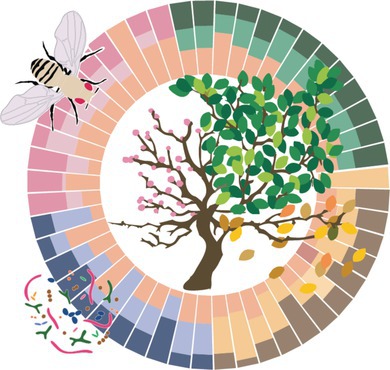

## 1 Introduction

Many insects associate with symbiotic microbes that enhance the hosts' fitness. While the insect provides a stable habitat for the associated microbes, the symbionts can provide a wide array of services to their host (Klepzig et al. 2009; Douglas 2015; Kaltenpoth et al. 2025). Symbiotic contributions to defence against antagonists, degradation of toxins, provisioning of limiting nutrients or breakdown of fastidious polymers are known to affect the health, growth and reproduction of their host (Itoh et al. 2018; Douglas 2014; Flórez et al. 2015; Hosokawa and Fukatsu 2020; Kaltenpoth et al. 2025). Microbial symbionts can be localised extra‐or intracellularly within host tissues, or in the host's immediate environment. The localization affects the symbionts’ transmission route and thereby impacts the stability of the association with the host as well as their exposure to abiotic factors. Microbial symbionts that reside intracellularly within host tissues, for example, in specialised organs called bacteriomes or in the hemolymph, are generally transmitted vertically through the germline and therefore sheltered from external disturbance (Bright and Bulgheresi 2010). By contrast, microbes found in the gut lumen or on the body surface of the insects usually experience a phase outside of the host during vertical transmission or are acquired de novo from the environment in every host generation (Garcia and Gerardo 2014; Douglas 2020; Ganesan et al. 2022; Salem et al. 2015). This exposure to abiotic factors and environmental microbes can strongly affect the composition, dynamics and functionality of host‐associated microbial communities (Itoh et al. 2019; Newell and Douglas 2014).

Many of the factors influencing the gut microbiota of insects, including diet, temperature and the influx of environmental microbes, vary throughout the year, especially in temperate regions. Consequently, insects can experience seasonal shifts in gut microbial communities (Šigutová et al. 2023; Hou et al. 2021; Li et al. 2022; Bleau et al. 2020) that can impact the host's fitness. For example, overwintering bark beetles ( *Dendroctonus valens* ) harbour a different gut microbiota than non‐overwintering beetles, which correlates with an increase in cold tolerance putatively enhancing the chances of survival during the overwintering period (Hou et al. 2021). In honeybees ( *Apis mellifera* ), the gut bacterium *Bartonella* is strongly enriched during the winter and shows the genomic capacity to recycle metabolic waste and produce essential amino acids, which may benefit host fitness during the period of nutrient scarcity (Li et al. 2022). However, seasonal changes in the microbial community can also be detrimental to the host, as exemplified by the higher sporulation of pathogenic fungi *Entomophthora muscae* during the cold season, which causes high mortality in *Musca domestica* (Watson and Petersen 1993). For most insects, however, the seasonal dynamics of their associated microbial communities remain unknown, despite the possible implications for host fitness.

Wild populations of *Drosophila melanogaster* are commonly found throughout seasons and harbour rich bacterial and fungal communities, since the saprophytic insect feeds on microbes growing on decaying fruits. Field studies characterising the gut microbes of the fruit fly showed that both deterministic factors and stochastic processes shape the variable microbiome (Adair and Douglas 2017; Henry and Ayroles 2022). Among the deterministic factors, the fruit fly diet (i.e., the fruit type) is described to have a strong impact on the structure and composition of the community along with the fly genotype. Indeed, providing a unique diet to different *Drosophila* species altered their gut microbiota, resulting in a convergence of bacterial communities among the different host species (Chandler et al. 2011). This dietary intervention effectively reduced the previously observed microbial divergence between host species (Chandler et al. 2011). Although the composition of the microbial community varies from one *D. melanogaster* individual to another, it is usually dominated by specific taxonomic groups, including acetic acid bacteria (*Acetobacteraceae*), lactic acid bacteria (*Lactobacillales*) and yeasts of the Saccharomycetaceae family (Wong et al. 2011; Wang and Staubach 2018). Laboratory studies showed that acetic acid bacteria ( *Acetobacter pomorum* ) and lactic acid bacteria ( *Lactobacillus plantarum* ) contribute to larval growth through activation of the TOR‐insulin signalling pathway (Storelli et al. 2018; Anagnostou et al. 2010; Shin et al. 2011), while dietary yeasts are essential to the insect development and nutrition by providing B vitamins, sterols, fatty acids and amino acids that are scarce in decaying matter (Broderick and Lemaitre 2012). Additionally, the overall microbial community has been shown to influence host behaviour (Markov et al. 2009), such as oviposition (Qiao et al. 2019; Kim et al. 2018), and it is promoting gut homeostasis by inducing a basal epithelial immune response and cell turnover (Buchon et al. 2013). While the association with microbes increases the fitness of the host, it is easily disturbed by thermal stress, as both cold and warm temperatures have been found to drastically change the composition of the fruit fly microbial community, with negative effects on the host fitness (Moghadam et al. 2018; Henry and Colinet 2018). Since diet and temperature are among the main factors changing throughout the seasons, *Drosophila melanogaster* and its associated microbes constitute an excellent system to study the seasonal dynamics of a symbiotic association.

While the microbial communities of wild *D. melanogaster* populations have been described across different geographic locations, altitudes and diets (Staubach et al. 2013; Martinson et al. 2017; Bost et al. 2018; Wang et al. 2020), less is known about temporal variation throughout the year in the fruit fly's microbial community, despite its potential relevance for host fitness. Recent studies have shown that bacterial communities in lab populations of *Drosophila melanogaster* are dynamic and influenced by temporal changes across seasons, as demonstrated by Henry et al. (2024) in a controlled field mesocosm setting. Building on this groundwork, our study extends these findings by examining the shifts in both bacterial and fungal communities of *Drosophila* populations in natural field settings from spring to summer, providing insights into how environmental conditions shape microbiome dynamics in nature. We aim at describing temporal patterns, over 5 months, in the microbial communities of three wild populations of *Drosophila melanogaster* located within the same latitude and altitude and in comparable habitats. Combining qualitative and quantitative approaches, we characterised the bacterial and fungal communities of 83 female fruit flies sampled throughout 5 months, covering winter, spring, and summer, revealing consistent temporal dynamics in bacterial and fungal community profiles and uncovering bioindicator microbial species as drivers of seasonality in the microbiota.

## 2 Material and Methods

### 2.1 Fly Collection

Fruit flies ( *Drosophila melanogaster* ) were collected in the apple ( *Malus domestica* ) orchard of the Bioobst Görnitz GmbH & Co. KG (51°08′58.1″ N 13°31′54.4″ E) near the city Meissen and next to a chokeberry ( *Aronia melanocarpa* ) orchard, in a cherry ( *Prunus avium* ) orchard surrounded by blackberries trees (*Rubus sect. Rubus*) near Pirna (50°56′54.9″ N 13°57′10.0″ E) and in the experimental field orchard of the Julius Kühn Institute, which features a diverse range of fruit species, including pome fruits like apples ( *Malus domestica* ), stone fruits such as cherries ( *Prunus avium* and *P. cerasus* ), plums ( *P. domestica* ) and berries (*Fragaria ananassa*) in Dresden‐Pillnitz (51°00′04.3″ N 13°52′53.4″ E, Figure S1). Collection took place once a month from April 2019 to August 2019. Flies were collected using insect aspirators (BioQuip Products, Rancho Dominguez, CA, USA), with one aspirator dedicated to each collection site to avoid cross‐contamination. For each sampling time point, a clean new air filter (BioQuip Products, Rancho Dominguez, CA, USA) was used with the aspirator to maintain sample integrity. The flies were directly aspirated into clean new plastic vials, immediately placed at sub‐zero temperatures in cooling boxes on crushed ice to immobilise them, and then transported to the laboratory. Upon arrival, the samples were stored at −80°C until further processing. Female *Drosophila melanogaster* were collected for all time points and locations except April and June in Pirna and April in Meissen. In total, 86 flies were selected, with the number of biological replicates for each collecting time point × site ranging from 5 to 16 individual flies. The samples were randomly assigned to six batches for DNA extraction, and the extraction batch was taken into account for the statistical analyses to control for possible artefacts arising from variation across DNA extractions.

### 2.2 Isolating DNA

Following Ridley et al. (2012), each fly was surfaced sterilised with a 4% sodium hypochlorite solution for 5 min and washed several times with sterile water. Individual flies were placed in liquid nitrogen and homogenised with a sterile pestle. Genomic DNA was extracted using the MasterPure complete DNA and RNA isolation Kit (Epicenter Technologies, Illumina Inc., San Diego, CA, USA) following the manufacturer's instructions including RNAse digestion. DNA extraction of a mock community standard (ZymoBIOMICS Microbial Community standard, Zymo Research, CA, USA) showed that both gram‐positive and gram‐negative bacterial DNA was well represented using this protocol. No‐template extractions were included to control for possible contamination. To counteract the overrepresentation of *Wolbachia* sequences in the bacterial community, a fraction of genomic DNA was digested with the restriction enzyme BstZ17I‐HF (New England Biolabs, Ipswich, MA, USA, catalog no. R0594S, 1 h incubation at 37°C) specifically cleaving the *Wolbachia* V1‐V2 region of the 16S rRNA gene, as demonstrated by Simhadri et al. (2017).

### 2.3 Host Barcoding

Following morphological identification, species assignment to *D. melanogaster* was additionally confirmed following the protocol described by Naserzadeh et al. (2020). A diagnostic PCR targeting a specific region of the cytochrome oxidase I (COI) gene was used to distinguish *Drosophila melanogaster* from morphologically similar species that may co‐occur in the wild, such as *D. simulans* . The PCR reaction mixture consisted of 1 μL template DNA, 1.25 μL of each specific primer (see Table S1 for primer sequences), 12.5 μL Q5 High‐Fidelity 2X Master Mix (New England Biolabs, MA, USA, catalog no. M0492S) and 9 μL nuclease‐free water. The reaction was prepared according to the manufacturer's instructions. Diagnostic PCRs were performed using a Mastercycler EP Gradient S Thermocycler (Eppendorf AG, Hamburg, Germany) with primer‐specific annealing temperatures (Table S1). Out of 83 collected samples, 82 were positively identified as *Drosophila melanogaster* . For the remaining sample, we were unable to amplify DNA using either the specific primers or general eukaryotic primers targeting a larger region of the COI gene. Despite the ambiguous PCR result, the microbial community profile of the unidentified sample clustered with samples from the same collection month and site. Additionally, it exhibited similar dominant taxa to the confirmed *D. melanogaster* samples. Based on these observations, and considering that our morphological identification was confirmed by the diagnostic PCR for all other 82 samples, we decided to retain this sample for further analysis, considering it most likely to belong to *Drosophila melanogaster* .

### 2.4 Bacterial, Fungal and *Wolbachia* Titres Quantification

Total bacterial and fungal titres were quantified as well as *Wolbachia* titres for each fly by real‐time quantitative PCR (qPCR). A total of 1‐μL template DNA was mixed with specific primers (0.5 μL for each primer, see Table S1 for primers sequences), qPCR SYBR Green Mix (10 μL, Thermo Fisher Scientific, Waltham, MA, USA catalog no. 4309155) and water (8 μL) following the manufacturer's instructions. qPCRs were run on a CFX Connect Real‐Time PCR Detection System (BioRad, Hercules, CA, USA, catalog no. 1855201) with primer‐specific annealing temperatures (see Table S1). Standard curves were used to determine copy numbers for each target and melting curves were analysed to check for amplified products specificity using the program Bio‐Rad CFX Manager 3.1(BioRad, Hercules, CA, USA).

### 2.5 Bacterial and Fungal Community Profiling

The bacterial V1‐V2 region of the 16S rRNA gene (after digestion of the *Wolbachia* 16S rRNA gene, see above) and the fungal ITS1 region were commercially sequenced by Starseq (Mainz, Germany) on an Illumina Miseq platform. The following primer combinations were used: 27F (5′‐AGAGTTTGATCMTGGCTCAG‐3′) and 338R (5′‐TGCTGCCTCCCGTAGGAGT‐3′) targeted eubacterial sequences (Simhadri et al. 2017), ITS1F (5′‐CTTGGTCATTTAGAGGAAGTAA‐3′) and ITS2 (5′‐GCTGCGTTCTTCATCGATGC‐3′) targeted fungal sequences (White et al. 1990; Gardes and Bruns 1993). Demultiplexed reads were analysed using R statistical Software v4.3.3 (Team 2013). Using Cutadapt v4.0 (Martin 2011), the fungal primers were removed. Using the R package DADA2 (version 1.26.0 (Callahan et al. 2016)), bacterial forward and reverse reads were custom trimmed to keep a length of 200 nucleotides. Fungal reads were trimmed to a minimum length of 50 base pairs. For both datasets, dereplication and chimera removal were performed using default parameters of DADA2. Bacterial and fungal amplicon sequence variants (ASVs) were then inferred, and each ASV was assigned taxonomically using the Silva classifier, v138.1 (Yilmaz et al. 2014) and UNITE classifier v9.0 (Abarenkov et al. 2024) to generate a bacterial and a fungal taxonomy table, respectively.

### 2.6 Statistical Analysis

All statistical analyses were performed on R, and each graph was generated with the *ggplot2* package (Wickham et al. 2016). Composition of the bacterial and fungal communities was analysed using the *Phyloseq* package (McMurdie and Holmes 2013). ASVs assigned to the Eukaryotic and Archaeal kingdoms as well as ASVs assigned to known intracellular endosymbionts of the fruit fly (*Wolbachia* and *Rickettsia* genus members) were removed from the bacterial taxonomy table. ASVs not assigned to the Kingdom Fungi were removed from the fungal taxonomy table. ASVs present simultaneously in the no‐template extraction and in the mock community sample, but absent from the theoretical mock community, were considered as contaminants, and ASVs previously known as human contaminant but absent from the fruit fly ecological niche were removed from the analysis. Bacterial and Fungal ASVs detected with a read count inferior to 3 and in less than 5.8% (5 replicates/86 samples = 0.058) of the samples were removed from the taxonomy tables used to generate the composition barplots for visualisation purpose, but they were kept in all other analyses (see Figure S2 for the bacterial and fungal communities' composition including the very rare ASVs). Differences in beta‐diversity were tested with permutational multivariate analysis of variance (PERMANOVA), *vegan* package (Oksanen et al. 2022) on Bray–Curtis dissimilarity matrices, and principal coordinate analyses (PCoA) were performed for visualisation using the *vegan* package. Differences in alpha diversity (Shannon index) and bacterial, fungal and *Wolbachia* titres obtained by qPCR were tested with linear mixed models, *lme4* package (Bates et al. 2015), with the collection month and site as fixed factors and the DNA extraction batch as a random factor. Pairwise comparisons were tested following the Tukey method with Benjamini–Hochberg correction with the *emmeans* package (Lenth et al. 2018). The indicator species analysis, performed with the package *indicspecies* (DeCaceres et al. 2016) aimed to identify microbial ASVs significantly associated with sampling time points. It provides a statistical value and a specificity index for each ASV/collection month pair (see Tables S2–S9). The Procrustes analysis, *vegan* package, was performed to compare the bacterial and fungal community structures and estimate the strength of a potential correlation between the two data sets through time. It provides dissimilarity and correlation indices as well as the percentage of variance accounted by the Procrustes fit. Both indicator species analyses and Procrustes analyses were performed for all sites combined and for each site individually in order to assess whether the temporal patterns in microbial community profiles were consistent across sites.

## 3 Results

### 3.1 The Bacterial Community of *D. melanogaster* Varies Across Seasons

**FIGURE 1:**
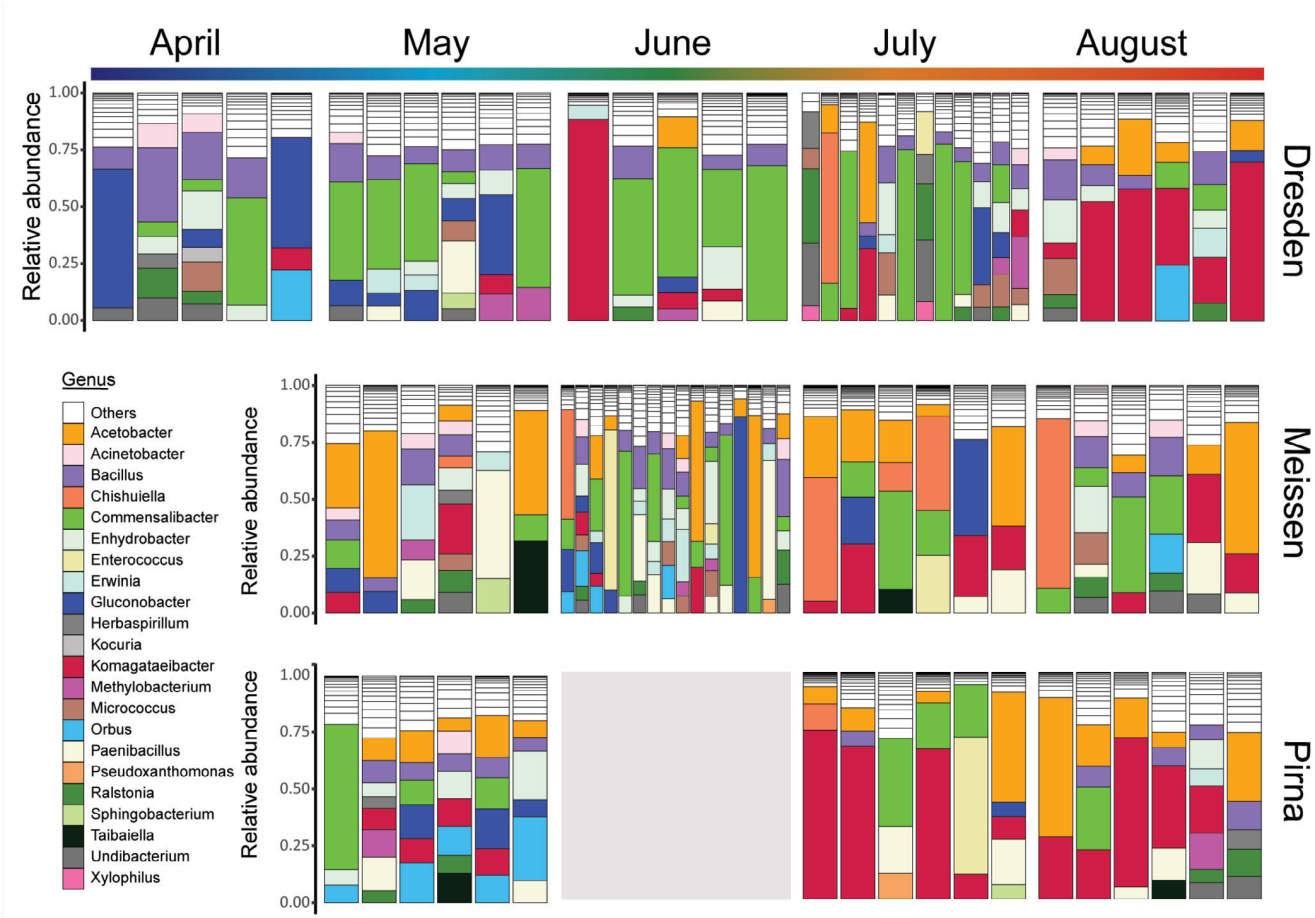
Bacterial communities of single *Drosophila melanogaster* individuals across 5 months and three different localities. Coloured sections of each bar show bacterial genera with a relative abundance above 5% in each sample. The rest of the ASVs were compiled in the ‘Others’ category.

The bacterial community of wild caught *Drosophila melanogaster* individuals was dominated by Alphaproteobacteria, mainly belonging to *Rhodospirillales*, as well as Bacilli bacteria (formerly Firmicutes) belonging to the *Bacillales*, and Gammaproteobacteria mainly belonging to the Enterobacteriales and Pseudomonadales (Figures 1 and S3 for bacterial community at the order level). Although the genus *Lactobacillus* was not visible in the top 33 genera dominating the bacterial community of the fruit flies, we found ASVs belonging to *Fructilactobacillus*, *Lactiplantibacillus* or *Levilactobacillus* (formerly combined in the genus *Lactobacillus*) in all collection site/time point combinations and in 83% of samples but in low relative abundances (below 5%).

An analysis of variance of the linear mixed‐effect models testing the effect of the collection month and site on the bacterial alpha‐diversity (Shannon index) and *Wolbachia* titres did not show any significant differences between the flies collected at different time points or at the different localities (LMEE, *p* values > 0.05, Tables S10 and S11, Figure S4). The same analysis on the bacterial titres showed a significant impact of the collection month, site as well as their interaction, but no consistent pattern was observed (LMEE, *p* values < 0.05, Tables S10 and S11, Figure S4).

**FIGURE 2:**
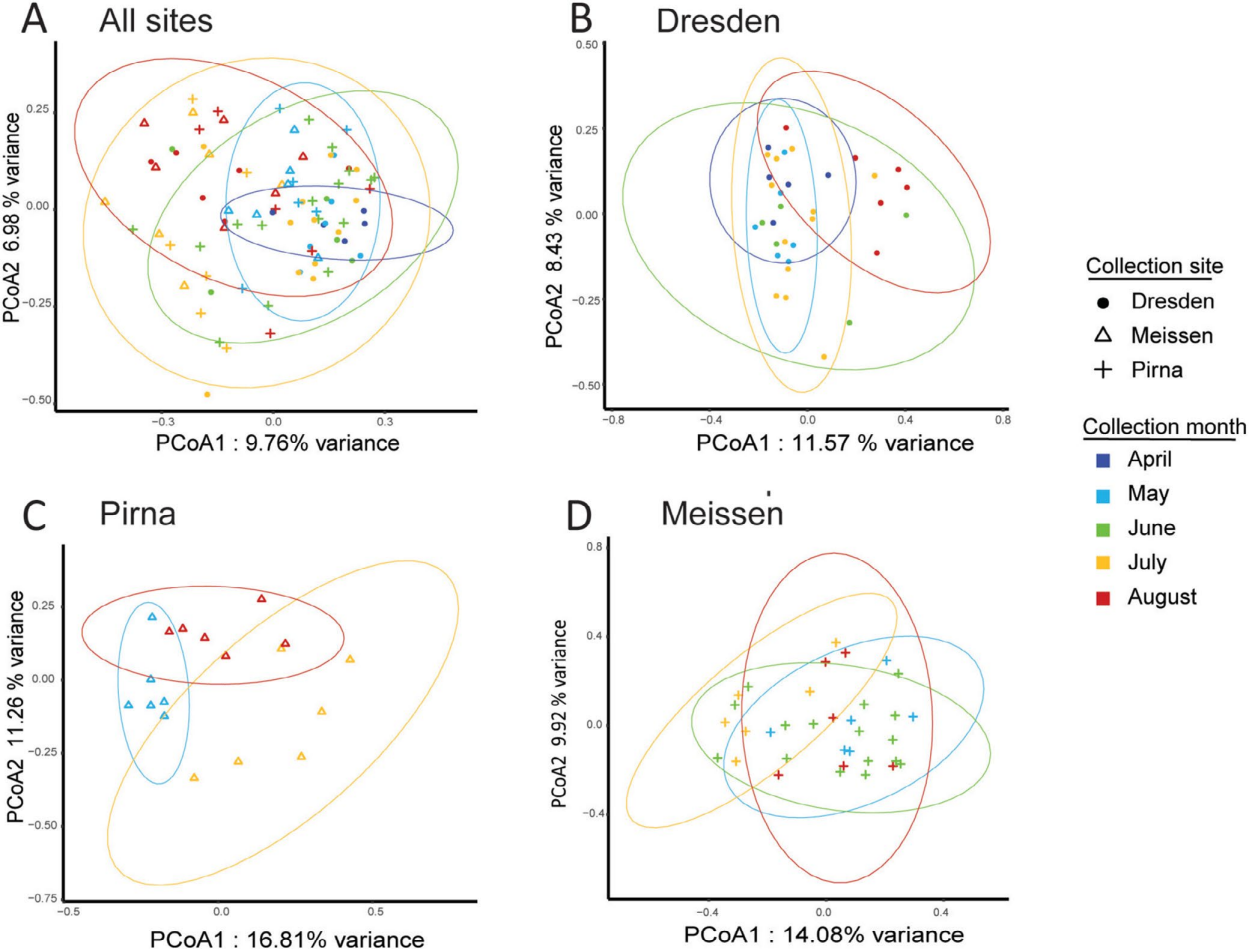
Differences in bacterial communities among wild‐caught *D. melanogaster* individuals from different collection months and sites. Principal coordinate analysis (PCoA) of Bray‐Curtis distances of (A) flies collected at all sampling sites and time points, (B) flies collected in Dresden, (C) flies collected in Pirna, (D) flies collected in Meissen. Each point, representing the bacterial community of a single fly, is coloured according to the sampling month, and shaped according to the sampling site. Ellipses (95% confidence interval) were added using the *ggplot2* package.

In order to identify patterns in the composition of the fly‐associated bacterial communities across sites and seasons, a Principal Coordinate Analysis (PCoA, Figure 2) was performed on Bray–Curtis distances along with permutational analysis of variance (PERMANOVA). Both the collection month (df = 4, *R* ^2^ = 0.07, *p* = 0.001) and the collection site (df = 2, *R* ^2^ = 0.04, *p* = 0.001), as well as their interaction (df = 5, *R* ^2^ = 0.08, *p* = 0.001) had a significant influence on the microbial community composition. This suggests that the bacterial community is differently shaped by the sampling month within each site. Indeed, the clustering of the flies' bacterial communities according to the sampling time is clearer in site‐specific PCoAs (Figure 2B–D) than in the overall PCoA (Figure 2A). Site‐specific PERMANOVAs show that the collection month is significantly structuring the bacterial communities within each site, with an explained variance ranging from 12% to 22% (Dresden: df = 4, *R* ^2^ = 0.16, *p* = 0.003, Pirna: df = 2, *R* ^2^ = 0.22, *p* = 0.001, Meissen: df = 3, *R* ^2^ = 0.12, *p* = 0.003). The DNA extraction batch had a significant impact on the microbial community only for Meissen samples, which was likely due to an overrepresentation of flies collected in June (Meissen: df = 6, *R* ^2^ = 0.18, *p* = 0.006, Pirna and Dresden: *p* > 0.05, Table S12) in Batch 6.

**FIGURE 3:**
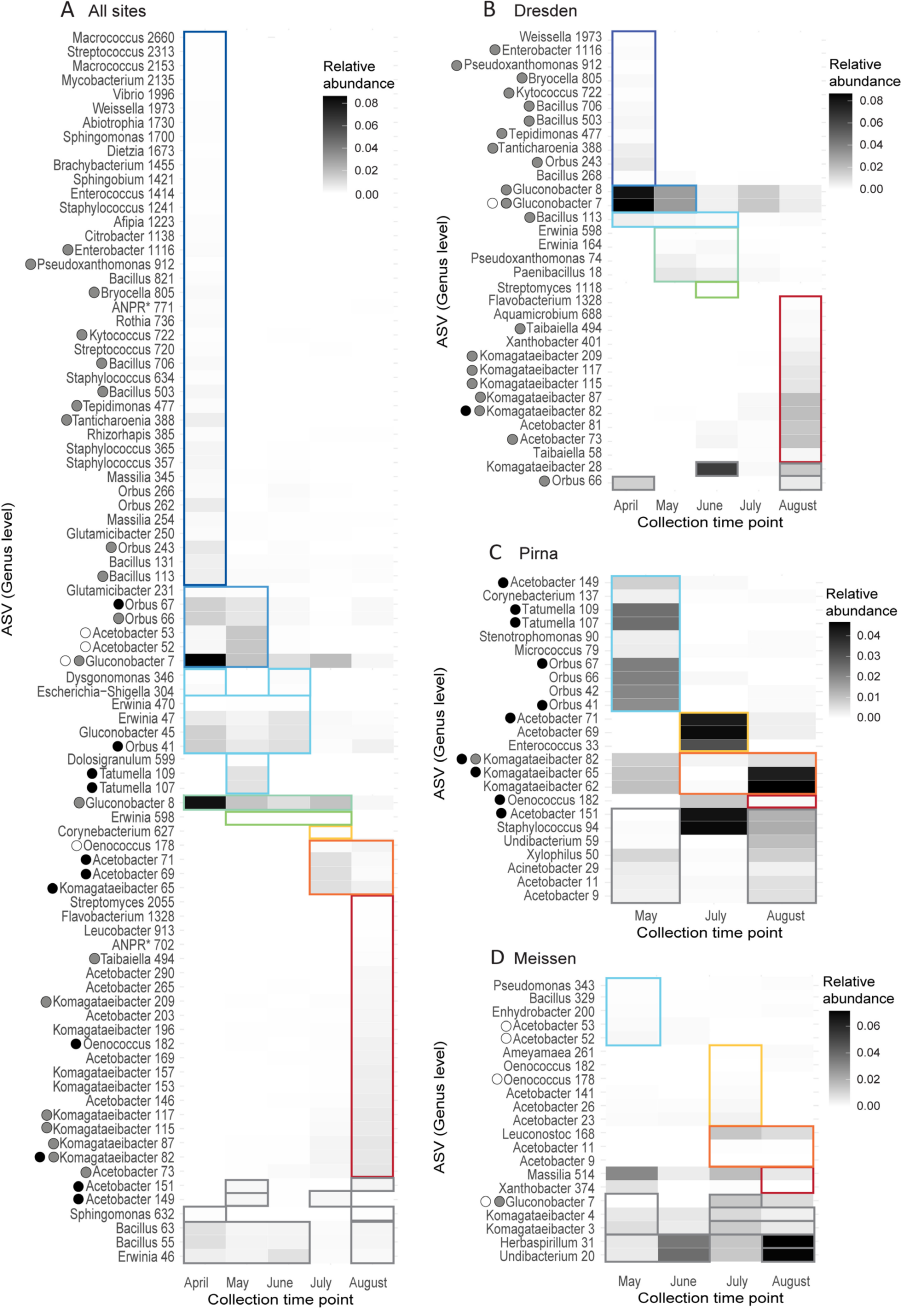
Bacterial indicator species for different sampling months in the microbial community of *D. melanogaster* (A) for all sampling sites combined, (B) for Dresden samples, (C) for Pirna samples, (D) for Meissen samples Each listed ASV is significantly associated to one or more collection months with a specificity coefficient > 0.40. The squares indicate which month(s) the ASVs are associated with, and the relative abundance of each ASV is given as a heatmap, with increasing darkness representing higher abundance. Dots in front of ASV names show in which data sets the ASV was identified as an indicator species; grey dots: indicator species in ‘all sites’ and Dresden, black dots: indicator species in ‘all sites’ and Pirna, white dots: indicator species in ‘all sites’ and Meissen.

To understand how the structure of the gut bacterial community was changing throughout the seasons, we performed an indicator species analysis to identify ASVs significantly associated with one or more sampling months (specificity coefficient: 0.39–1 and *p* value < 0.05, see Tables S2–S5) for all sites combined (Figure 3A) and for each site individually (Figure 3B–D). Comparing the indicator species from all sites to the ones associated with each individual site allowed us to see if patterns are consistent across sites. Among the bacterial ASVs, 86, 33, 21 and 24 were significantly associated with one to four collection months in ‘all sites’ together, Dresden, Pirna, and Meissen, respectively. Briefly, 20, 10 and 4 of these ASVs were found in ‘all sites’ plus either Dresden, Pirna or Meissen, respectively (grey, black and white circles in Figure 3). Two ASVs were identified as indicator species for ‘all sites’ and two individual sites: *Gluconobacter* 7 in Dresden and Meissen, and *Komagataeibacter* 82 for Dresden and Pirna. At the genus level, more ASVs belonging to the same genera as the aforementioned ones were shared between their individual site and ‘all sites’. ASVs belonging to the *Komagataeibacter* genus were consistently associated with the months of July and/or August for each of the sites, *Orbus* ASVs were found in April or May, and *Gluconobacter* ASVs were found in April and May. Indeed, we found 6/33 and 3/21 indicator ASVs from Dresden and Pirna, respectively, belonging to the genus *Komagataeibacter*, and 1/33 and 4/33 ASVs from Dresden and Pirna, respectively, belonging to the genus *Orbus*. The indicator species analysis conducted on samples from all sites combined highlighted 54/86 ASVs not detected in the site‐specific analysis. This means that while they were not present and abundant enough in specific sites, at the larger scale they were found as indicator species among the different sites in specific months. Interestingly, out of those ASVs, we found two *Orbus* ASVs as indicators for April, one *Gluconobacter* ASV as an indicator for April–May–June, and three *Komagataeibacter* ASVs as indicators for August. Other ASVs belonging to the genera *Erwinia* and *Oenococcus* were detected in a similar manner in April to July and July to August, respectively. Sixteen *Acetobacter* ASVs were found as indicator species in all sites, combined or individually, but not consistently associated with the beginning or end of the collecting period. No ASVs belonging to *Commensalibacter* were identified as indicator species for specific months since it is found as a dominant taxon of the bacterial community across all sampling sites and time points (Figure 1). With this analysis, we identified bacterial candidates for the coldest and warmest months, which differed in their identity. Indicator species for the colder spring months were among bacteria belonging to the genera *Gluconobacter* and *Orbus*, whereas indicator species for warmer summer months belonged to the genus *Komagataeibacter*. Specificity coefficients and statistical values are available in Table S2. Thus, while Rhodospirillales occurred consistently in samples from all collection months and locations, they were represented by different genera across the sampling months: While *Acetobacter* and *Commensalibacter* species were found across the whole sampling period, *Gluconobacter* and *Komagataeibacter* species seemed to be among the dominant genera in early or late months, respectively (see also Figure 1). There seems to be a site‐specific strain diversity in these seasonal patterns.

### 3.2 The Fungal Community of *D. melanogaster* Varies Across Seasons

**FIGURE 4:**
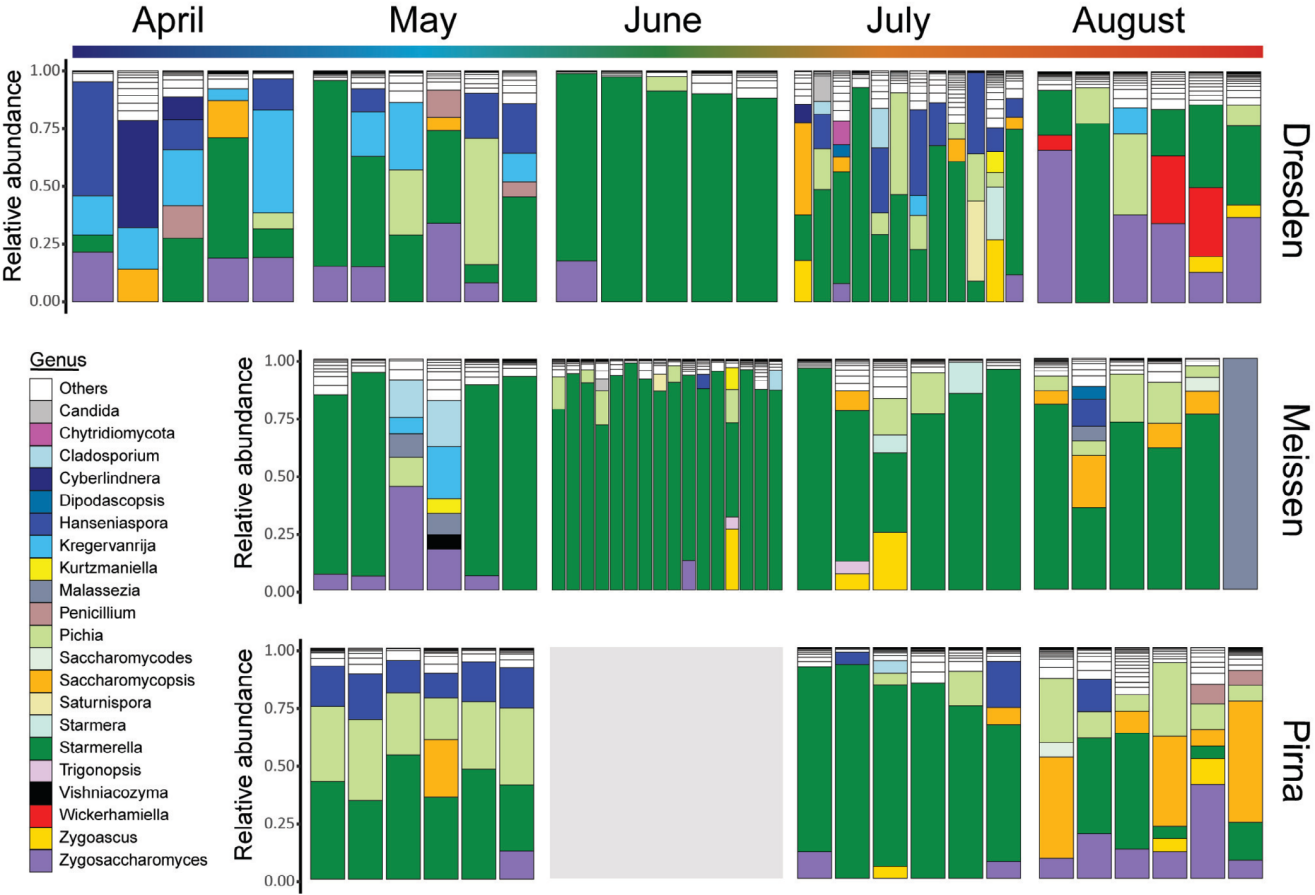
Fungal community of single *Drosophila melanogaster* individuals. Coloured sections of each bar show fungal genera with a relative abundance above 5% in each sample. The rest of the ASVs, agglomerated in rarer genera, were compiled in the ‘Others’ category.

As described previously, the fungal community associated with *D. melanogaster* was dominated by yeasts from the Saccharomycetales order (Ascomycota, Saccharomycotina, Saccharomycetes, see Figure S5). In particular, *Starmerella* species were abundant in all sampling sites and collection months (Figure 4). Depending on the sampling site and collection month, yeasts belonging to *Saccharomycopsis*, *Pichia*, *Hanseniaspora*, *Kregervanrija* and *Zygosaccharomyces* genera were found among the 21 most dominant genera (Figure 4).

The alpha‐diversity (Shannon index) of the fungal community significantly varied across months and sampling locations, as well as their interaction (LMEE, Table S10). Pairwise comparisons between months revealed significant differences in alpha‐diversity for each site, with the fungal diversity consistently decreasing to reach a minimum in June or July and then increasing to reach similar levels of alpha‐diversity in August as in the spring months (Figure S4). The absolute fungal titres only differed between collection months for samples collected in Meissen, and only the interaction of the collection month and site had a significant impact (LMEE, Tables S10 and S11, Figure S4).

**FIGURE 5:**
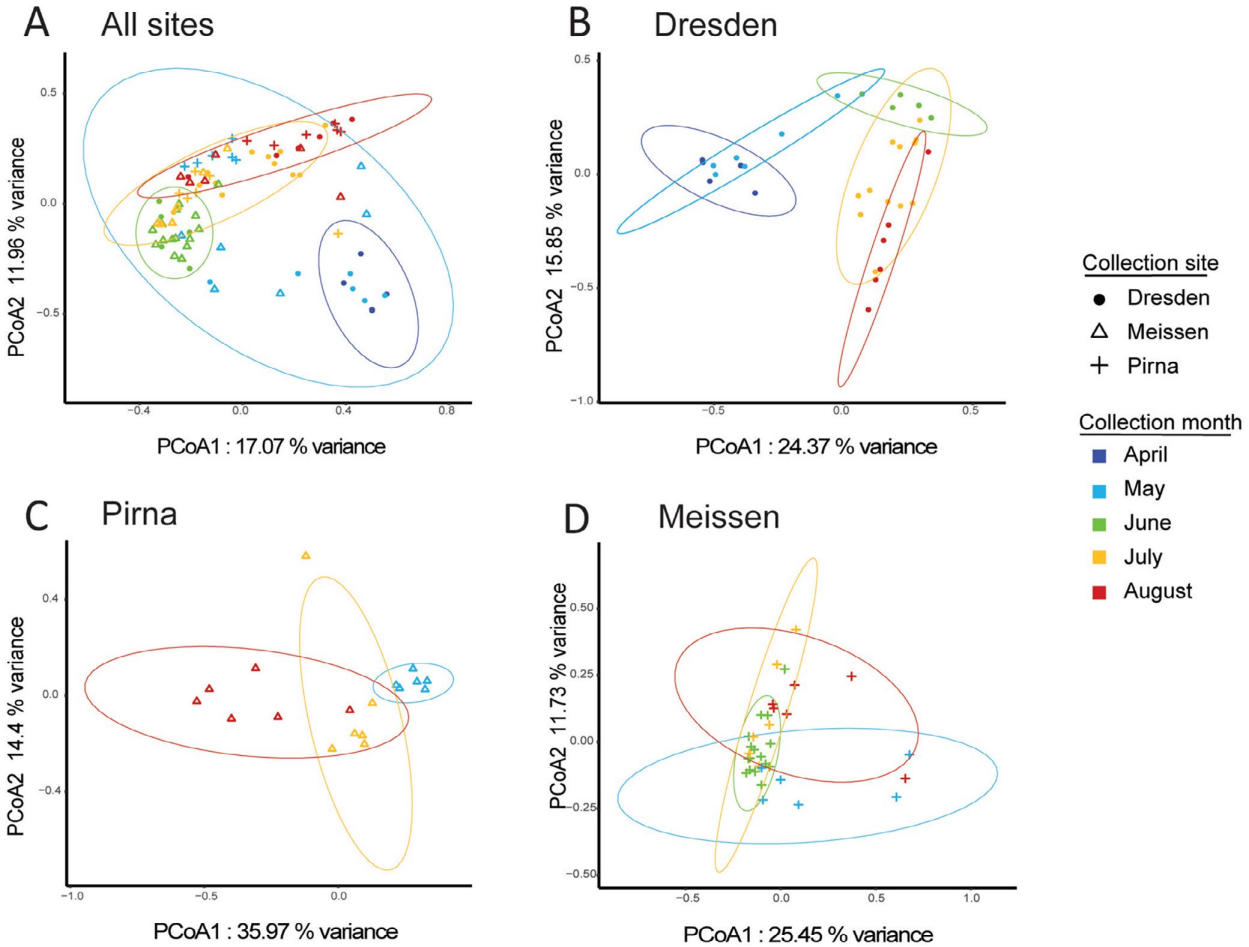
Differences in fungal community profiles among wild‐caught individuals from different sampling months and sites. Principal coordinate analysis (PCoA) of Bray‐Curtis distance (A) of flies collected at all sampling sites and time points, (B) of flies collected in Dresden, (C) of flies collected in Pirna, (D) of flies collected in Meissen. Each point, representing the bacterial community of a single fly, is coloured according to the sampling month, and shaped according to the sampling site. Ellipses (95% confidence level) were added using the *ggplot2* package.

PERMANOVAs and PCoAs based on Bray–Curtis distance matrices revealed that the *D. melanogaster* ‐associated fungal community was shaped by the collection month (df = 4, *R* ^2^ = 0.20, *p* = 0.001), the collection site (df = 2, *R* ^2^ = 0.10, *p* = 0.001), and the interaction of both factors (df = 5, *R* ^2^ = 0.12, *p* = 0.001). Within each site, the collection month was also structuring the fungal community, with a greater variance explained (23%–42%) than for the bacterial communities (Dresden: df = 4, *R* ^2^ = 0.41, *p* = 0.001, Pirna: df = 2, *R* ^2^ = 0.42, *p* = 0.001, Meissen: df = 3, *R* ^2^ = 0.23, *p* = 0.001). Correspondingly, the PCoAs showed the fungal community clustering according to the different months within each site (Figure 5B–D) as well as in the overall PCoA. The DNA extraction batch did not have any effect on the fungal community composition (*p* > 0.05, Table S12).

**FIGURE 6:**
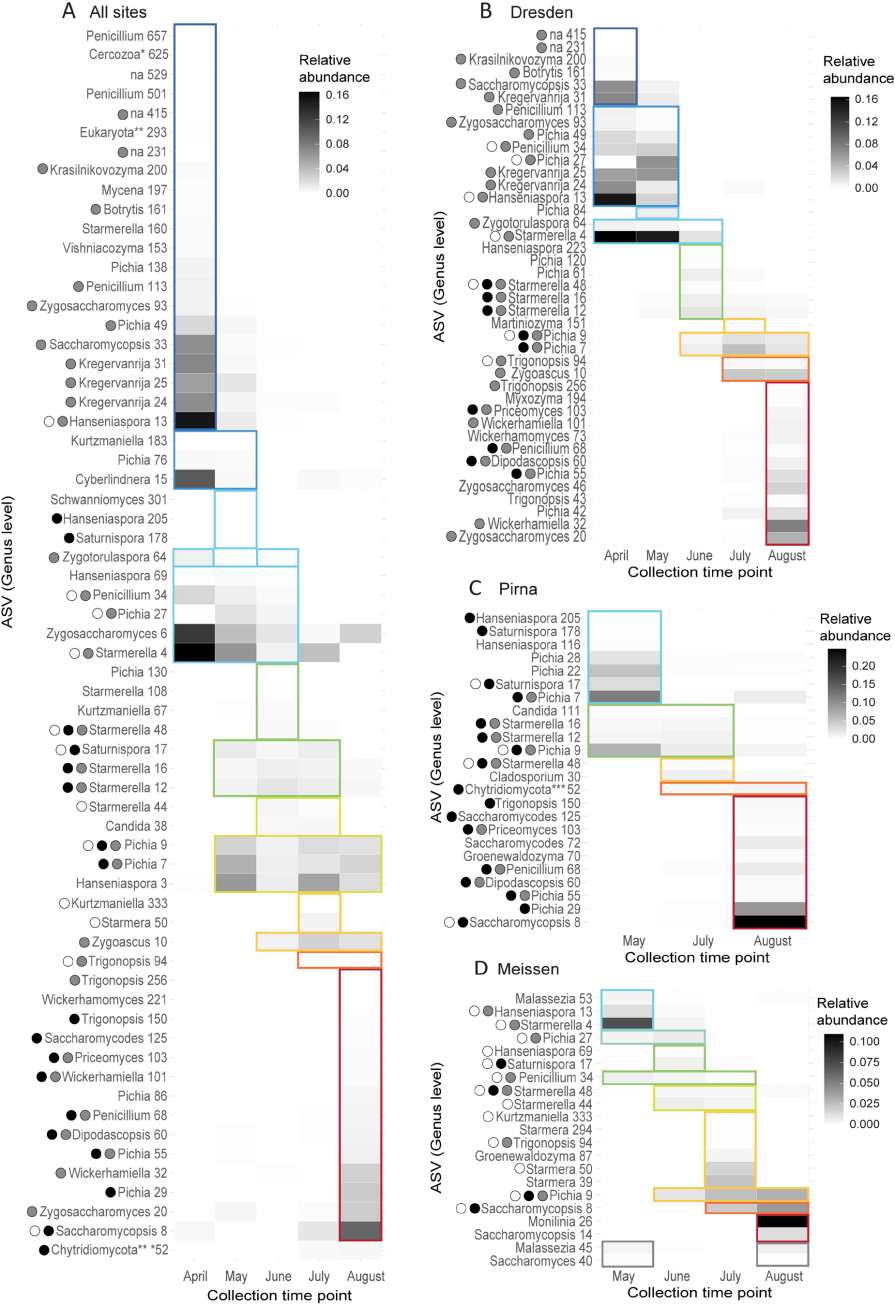
Fungal indicator species for different sampling months in the microbial community of *D. melanogaster* (A) for all sampling sites combined, (B) within Dresden samples, (C) within Pirna samples, (D) within Meissen samples each ASV was significantly associated to one or more collection months with a specificity coefficient above 0.40. The squares indicate which month(s) the ASVs is associated with. The mean relative abundance for each ASV within a collection point was calculated with the *phyloseq* package. Dots in front of ASV names show in which data sets the ASV was identified as an indicator species; grey dots: indicator species in ‘all sites’ and Dresden, black dots: indicator species in ‘all sites’ and Pirna, white dots: indicator species in ‘all sites’ and Meissen.

The fungal indicator species analysis (Figure 6) revealed 67, 41, 24 and 20 ASVs significantly associated (specificity coefficient: 0.41–1 and *p* value < 0.05, see Tables S6–S9) with one or more sampling months in all sites combined, Dresden, Pirna, or Meissen, respectively. 31/41, 18/24 and 12/20 ASVs were found in ‘all sites’ plus either Dresden, Pirna or Meissen, respectively (grey, black and white circles in Figure 6), which represent more than half of the indicator species found in the individual sites. Two ASVs (*Starmerella* 48 and *Pichia* 9) were identified as indicator species for all sites combined and for each of the sites individually. Fifteen ASVs were identified as indicator species for ‘all sites’ and two individual sites: Five were indicator species in all sites combined plus Dresden and Meissen, eight ASVs in all sites combined plus Dresden and Pirna, and two ASVs were indicator species in all sites combined plus Pirna and Meissen. The indicator species analysis conducted on samples from all sites combined highlighted 22/67 ASVs not detected in the site‐specific analysis. In contrast to the bacterial indicator species, some of the genera highlighted in this analysis were significantly associated with both early and late collection months. However, most ASVs within a genus were specifically associated with either early or late collection months, showing that it is at the strain level that we can see a temporal pattern. For example, a total of 16 *Pichia* ASVs were found as indicator species in specific sites and/or in all sites combined. Focusing on the ASVs found in at least two analyses, we found *Pichia* 49 in April to June (April in ‘all sites’, April–May in Dresden), *Pichia* 27 in April to June (April–May–June in ‘all sites’, April–May in Dresden and May–June in Meissen), *Pichia* 55 in August (in ‘all sites’, Dresden and Pirna) similarly to *Pichia* 29 (in ‘all sites’ and Pirna) and *Pichia* 7 and 9 showed inconsistent patterns across sites. The 12 other ASVs were not shared between analyses and showed collection month specificity at the species level. Table S13 shows the indicator species, at the species level and for all analyses combined, associated with one to three consecutive months with the exception of *P. eremophila* and *P. kluyveri*, which correspond to *Pichia* 9 and 7. Regarding indicator species belonging to the genus *Hanseniaspora* (six in total), *Hanseniaspora* 13 was found in April to May (April in ‘all sites’, April–May in Dresden and May in Meissen) and *Hanseniaspora* 205 was found only in May (‘all sites’ and Pirna). At the species level, there was a clear pattern as each month was associated with different *Hanseniaspora* species. While we also observed specific strain‐month associations within the genus *Starmerella*, all ASVs highlighted belonged to *S. stellata* . We also observed strain and species‐specific temporal patterns for ASVs belonging to *Kurtzmaniella*, *Candida*, *Penicillium*, *Zygosaccharomyces*, *Saccharomycopsis and Trigonopsis*. Finally, ASVs belonging to a unique strain within a genus were associated with specific collection months across the different analyses: seven genera (*Botrytis*, *Eukaryota*, *Cercozoa*, *Krasilnikovozyma*, *Mycena*, *Vishniacozyma* and an unknown genus) were only associated with the month of April, one genus (*Schwanniomyces*) with the months of April and May, two genera (*Cyberlindnera*, *Kregervanrija*) with the month of May, one genus (*Zygotorulaspora*) with the months of April, May and June, one genus (*Saturnispora*) with the months of May, June and July, one genus (*Zygoascus*) with the months of June, July and August, three genera (*Cladosporium*, *Martiniozyma*, *Starmera*) with the month of July, one genus (*Groenewaldozyma*) with the month of July and August, and seven genera (*Dipodascopsis*, *Wickerhamomyces*, *Monilinia*, *Myxozyma*, *Priceomyces*, *Saccharomycodes*, *Wickerhamiella*) with the month of August.

In summary, we were able to identify ASVs as fungal indicators for colder months and other ASVs as fungal indicator species of warmer months. Interestingly, while we found ASVs belonging to the genera *Pichia* and *Starmerella* across all months, the ASVs representing these genera differed across months, indicating temporal changes in the fungal community at the strain level.

### 3.3 Correlation of the Bacterial and Fungal Communities Through Time

The Procrustes analysis comparing bacterial and fungal community structures revealed a significant (*p* = 0.001) correlation of 0.29 with a high dissimilarity value of 0.91 suggesting that, while the bacterial and fungal communities have very different overall structures, there is still a moderate but consistent correlation between them across sampling months (Figure S6A). Performing the same analysis for each site individually revealed significant correlations between fungal and bacterial communities for Dresden and Pirna, but not for Meissen (Table S14, Figure S6B,C,D). Dissimilarity indices remained high in all individual sites (0.71 to 0.93).

## 4 Discussion

Seasonally changing abiotic and biotic factors significantly impact the exposure to microbial communities for animals living in temperate environments. Here, we showed that the bacterial and fungal communities of three wild populations of *Drosophila melanogaster* were changing from early spring to late summer across three different populations in Germany. This temporal effect is driven by changes in the presence and relative abundance of specific bacterial and fungal indicator species. By contrast, alpha‐diversity and total microbial load as well as the abundance of the intracellular symbiont *Wolbachia* were not following a seasonal pattern in these natural populations of *D. melanogaster* .

The bacterial communities of *D. melanogaster* are known to be shaped by both deterministic and stochastic processes (Adair and Douglas 2017; Henry and Ayroles 2022). While seasonality has not previously been the main focus of a field bacterial profiling effort in *D. melanogaster* , biotic and abiotic factors changing over the seasons have been described as impacting the bacterial communities of the fruit fly in the field across different continents. Specifically, diet (Chandler et al. 2011; Staubach et al. 2013) and annual temperatures (Wang et al. 2020) have a strong influence on the structure of *Drosophila melanogaster* bacterial communities as well as those of other drosophilids. Bacterial communities of flies collected from different geographic locations along latitudinal, longitudinal or altitudinal gradients exhibit distinct differences (Henry and Ayroles 2022; Wang and Staubach 2018; Staubach et al. 2013; Martinson et al. 2017; Bost et al. 2018; Wang et al. 2020; Adair et al. 2018). Each location's specific climate and ecology, which includes differences in temperature and vegetation (substrate for *D. melanogaster* ), may partially explain the observed bacterial community. Interestingly, these geographic patterns in bacterial communities may be analogous to the effects of seasonality, as each season is characterised by different mean temperatures and changes in vegetation. In some studies that form the foundation of bacterial community research in fruit flies, the sampling date, though not the primary focus, was included in the statistical models. Analysing bacterial compositions at different time points reveals patterns similar to our findings. For instance, Behrman et al. (Behrman 2017) found *Gluconobacter* predominantly in flies collected in autumn (October) while Henry and Ayroles (2022) identified *Komagataeibacter* in wild‐caught flies throughout the year, except for the early fall months (September–October). Our indicator species analysis results align with these previous findings, detecting *Gluconobacter* in colder months and *Komagataeibacter* in warmer months. Similar to the temporal shifts in bacterial communities, we also detected consistent and strong changes in the yeast community associated with *D. melanogaster* across the sampling months. While our findings corroborate recent studies (Quan and Eisen 2018; Valer et al. 2016; Chandler et al. 2012) showing that fungal communities are primarily composed of Ascomycetes yeasts and appear to be influenced by the insect's diet, the impact of sampling date on fungal community composition has, to our knowledge, not been investigated previously. Finally, despite the consistent effect of the sampling months, we observed significant differences in the bacterial and fungal communities of flies across the different sites. Even though all collecting sites were orchards with similar species of fruit trees, feeding opportunities likely varied across the sites, possibly resulting in different dietary intakes of the flies. This, together with stochastic processes shaping the flies’ microbial communities (Adair and Douglas 2017) might explain the differences between sampling sites as well as the considerable inter‐individual variation in the microbial communities within each site and month.

As a multivoltine species living in temperate climates, *Drosophila melanogaster* has to survive throughout the different seasons (Behrman et al. 2015). Adults are known to enter a dormant state at the onset of winter in response to colder temperatures and shorter photoperiods (Saunders et al. 1989). While other drosophilids only appear late in spring, *Drosophila melanogaster* has a wide range of viable temperatures (between 12°C and 29°C) allowing it to exit diapause when spring begins. Populations reach their maximum size in July, followed by a decreased but subsequently constant level through the end of summer and fall (Gleason et al. 2019). The insect needs to have a flexible diet to find resources all year long, especially since its main substrates are rotting fruits, a highly seasonal and ephemeral resource. In 1954, Yoshimoto (1954) found *Drosophila melanogaster* on a wide array of decaying substrates including apples, grapes, pears, peaches, bananas, persimmon, pokeweed, tomatoes, as well as cucumbers, green peppers and mushrooms, covering diets available across different seasons. The dietary shifts across the seasons bring about changes in macronutrient availability and confront the flies with different microbial communities growing on the respective substrates. Therefore, the changes in microbial communities of *Drosophila melanogaster* that we observe might be caused by seasonal differences in the microbial communities that the fruit flies ingest with the diet or result from dietary differences in nutrient availability shaping the nutritional environment in the gut. Collection efforts throughout several years are necessary to confirm that the temporal patterns we observed are indeed shaped by seasonality.

While the environment certainly shapes the microbial communities of *Drosophila melanogaster* , the host genotype also plays a significant role (Henry and Ayroles 2022). Along with host genotype‐driven variation in metabolic processes (Broderick and Lemaitre 2012), behavioural traits (Chandler et al. 2011), health and longevity (Ren et al. 2007) and host immunity (Erkosar and Leulier 2014) modify the interactions with microbes, determining the success of microbial establishment in the host. Indeed, while the gut of *D. melanogaster* is exposed to transient microbes passing with food intake, its colonisation is regulated by the insect immune system, particularly via the release of anti‐microbial peptides (AMPs) (Broderick 2016; Marra et al. 2021; Alarco et al. 2004), the activation of Duox enzymes (Ha et al. 2005), and the adjustment of the pH in the midgut region (Overend et al. 2016), as well as microbe‐microbe interactions (Itoh et al. 2019). Interestingly, Behrman et al. (2018) found significant seasonal variation in immune responses of *D. melanogaster* , which changed rapidly within about 10 generations from spring to autumn. This was due to seasonal changes in allele frequencies of drosomycin genes, leading to varying immune responses to infections by two natural pathogens. This rapid cyclic response to shifts in microbial exposure may be an evolutionary strategy to mitigate seasonal biotic stressors. Finally, abiotic factors changing with seasonality can trigger specific immune responses in *D. melanogaster* . For example, an acute cold‐shock triggered immune activation such as an increase in hemocytes circulating in the hemolymph as well as Turandot‐A and diptericin expression, while the clearance of gram‐positive bacteria was impaired (Salehipour‐Shirazi et al. 2017). In the red flour beetle, *Tribolium castaneum* , a thermal shock experienced by the parents even resulted in increased immunity and differential developmental rates of the offspring, with specific responses for parental exposure to heat and cold, respectively. Presumably, there is a cross‐talk between thermal tolerance and immunity where thermal shock acts as a cue allowing the flies to anticipate future stressful environmental conditions (Sinclair et al. 2013). In summary, while the impact of abiotic factors on immunity is well established in *D. melanogaster* and other insects, its implications for microbial community composition currently remain largely unknown.

While environmental and genotypic variation influences the *Drosophila*‐associated microbial community, this intraspecific variation may in turn affect the individual fly's fitness (Gleason et al. 2019). Indeed, insect‐associated microbes can expand or constrain their host's ability to withstand changing abiotic factors like changing temperature and humidity (Lemoine et al. 2020) or biotic challenges such as pathogen infections (Flórez et al. 2015) or nutrient‐poor diets (Cornwallis et al. 2023), which are factors changing across seasons. In the mountain pine beetle‐fungi symbiosis, the insect hosts three fungal species essential for bark colonisation, whose seasonal abundances peak at different optimal temperatures (Rice et al. 2008). The seasonal dynamics in fungal associates allows the beetles to cope with temperature changes throughout the seasons and therefore appears to benefit host fitness. In aphids, the prevalence of *Hamiltonella defensa* is influenced by environmental factors such as temperature, which can impact its protective efficacy against parasitoids (Oliver and Higashi 2019; Smith et al. 2021; Gimmi et al. 2023). While *H. defensa* provides resistance to parasitoids, its effectiveness can be compromised under high temperatures, suggesting that seasonal variation in temperature may modulate the symbiont's protective benefits against parasitoids (Oliver and Higashi 2019; Smith et al. 2021). The seasonal dynamics of parasitoid wasps, which often peak during warmer months, may interact with temperature‐driven changes in *H. defensa* prevalence, potentially affecting the overall protection conferred to aphids against these seasonal threats (Smith et al. 2021; Wu et al. 2025). In honey bees ( *Apis mellifera* ), a shift in the microbial community during winter reveals that *Bartonella*, a bacterium found dominant only in winter populations, may efficiently recycle metabolic waste into pyruvate and is the only member of the winter microbial community that produces essential amino acids, tryptophan and phenylalanine, which are not available in the winter diet (Li et al. 2022). These examples showcase that seasonal variation in microbial communities can affect insect performance and population dynamics, supporting Lange et al.'s (2023) statement that ‘the right combination of microbial partners may support a resilient population, while a shift can contribute to population decline or invasion’.

In *D. melanogaster* , there is no clear evidence yet that the association with the gut microbial community facilitates seasonal adaptation in nature. However, under laboratory conditions, it is established that the insect relies heavily on its microbial partners to survive and develop in the face of nutrient scarcity (McMullen II et al. 2020), fluctuating temperatures (Henry and Colinet 2018) and the presence of pathogens (Teixeira et al. 2008). Additionally, Brankatschk et al. (2018) demonstrated that the diet (plant‐derived or yeast‐derived) significantly influences *D. melanogaster's* thermal optimum, with severe consequences for the insect's fitness, providing evidence for a diet shift as a potential strategy to cope with the challenges of seasonality. Our results indicate that a seasonal substrate shift experienced by fruit flies in nature could be accompanied by a shift in microbial communities. Specifically, we observe temporal changes in the bacterial community, with distinct indicator species present in early spring and late summer. At the genus level, we see a shift in the predominant Rhodospirillales in the bacterial community from April to August, with *Gluconobacter oxydans* being the main representative in the earlier months, while *Komagataeibacter* species dominate the family in later months. While both bacteria belong to the acetic acid‐producing group, along with *Acetobacter* bacteria providing essential metabolites to the host (Crotti et al. 2016), they are phylogenetically distinct groups with differences in their physiologies and habitat (Matsutani et al. 2011). While both are able to oxidise ethanol, *Komagataeibacter* is more efficient than *Gluconobacter* at high ethanol concentrations (Guillamón and Mas 2009). Conversely, *Gluconobacter* can utilise a wider range of substrates in addition to ethanol, such as many sugars, sugar alcohols and glycerol (Matsushita and Matsutani 2016). Since fruits have higher ethanol concentrations in late summer due to advanced decomposition by yeasts (Morais et al. 1995), an increased abundance in *Komagataeibacter* may be beneficial to the flies by reducing ethanol content and increasing the availability of acetic acid. Whether *Gluconobacter* utilisation of other substrates confers an advantage to the host in early spring remains to be elucidated.

Connecting the temporal changes observed for fungal community profiles to possible implications for the fly's fitness remains challenging. The yeasts found in fruit fly communities typically inhabit rotting fruits and are known for their fermentation abilities, and they have been described as essential for *Drosophila melanogaster* nutrition, providing essential amino acids and vitamins, impacting the host’s overall fitness (Anagnostou et al. 2010; Stefanini 2018; Meshrif et al. 2016). However, their specific interactions with the insect remain poorly understood, and it is still unclear whether they actively interact with the insect's gut or passively release nutrients to the gut lumen, for example, due to lysis of a part of the population (Günther and Goddard 2019). Our results reveal clear temporal patterns across seasons in the yeast community for the three fruit fly populations. *Starmerella* yeasts, known to attract *D. melanogaster* and modify its foraging behaviour, are consistently found in most samples and collection months, seemingly dominating the fungal community. *Kregervanrija* yeasts are only present in early months, and other genera appear only at the beginning of spring and the end of summer (e.g., *Pichia*, *Hanseniaspora*, *Saccharomycopsis*, *Zygosaccharomyces*). While these genera can be found simultaneously as early and late indicator taxa, strain‐level analysis indicates distinct early and late species within each genus (Table S13). Whether these taxonomic differences confer fitness advantages to the flies in the seasonal context remains to be determined, as current knowledge primarily focuses on fermentative abilities under different conditions (including temperature) and genetic divergences among these species. Further experimental work conducted on gnotobiotic flies infected with naturally associated yeasts exposed to different temperatures and diets is necessary to unravel potential fitness implications of interindividual and seasonal variation in fly‐associated yeast communities.

While we observed no consistent temporal pattern in *Wolbachia* abundance across sites, Henry et al. (2024) reported *Wolbachia*‐driven seasonal modulation of bacterial communities in controlled mesocosms. This contrast likely originates from methodological differences: their experimental design isolated *Wolbachia*'s effect through artificial population manipulations, whereas our study reflects natural *Wolbachia* dynamics in wild flies exposed to fluctuating environmental conditions. The absence of *Wolbachia* titre changes in our field populations suggests that its influence may be context‐dependent, potentially overshadowed by stronger drivers like dietary shifts in natural settings.

In conclusion, our study demonstrates temporal shifts in the bacterial and yeast communities associated with *Drosophila melanogaster* across months. These changes in community structure and composition may result from either the changing microbial environments that the flies encounter, including changes in diet, or selective recruitment by the flies themselves. While microbes are known to enhance fly fitness, further research is needed to determine if intraspecific variation in these microbial communities leads to fitness differences, particularly whether the temporal shifts across seasons provide any specific fitness benefits. Understanding the adaptive significance of seasonal dynamics in insect‐associated microbial communities could shed light on the role of microbes in expanding the ecological niche of their hosts and their contribution to the geographic range expansion of *D. melanogaster* from the tropical ancestral sub‐Saharan range to the colonisation of multiple continents with temperate climates (Lachaise and Silvain 2004).

## Author Contributions

**Marion Margaux Lemoine:** conceptualization, methodology, investigation, data curation, formal analysis, visualization, writing – original draft, writing – review and editing. **Thomas Wöhner:** resources, writing – review and editing. **Martin Kaltenpoth:** conceptualization, supervision, project administration, writing – review and editing, writing – original draft, funding acquisition, resources.

## Conflicts of Interest

The authors declare no conflicts of interest.

## Supporting information


Data S1.

## Data Availability

The datasets presented in this study are deposited in online repositories. The BioProject PRJNA1148441 on NCBI contains the amplicon sequences corresponding to the bacterial and fungal communities. The ASV and taxonomy tables as well as the sample metadata are available on Edmond (the Open Research Data Repository of the Max Planck Society), https://doi.org/10.17617/3.QUGYBW.
