# Supplementary material for: Microbial Community Dynamics in Natural Drosophila melanogaster Populations Across Seasons
Source: Environ Microbiol. 2025 Jun 2;27(6):e70104. doi: 10.1111/1462-2920.70104 (PMC12129609; doi:10.1111/1462-2920.70104)
Supplement: Supplementary file 1 — Data S1. [file EMI-27-e70104-s001.pdf]

Appendix

for

Seasonal shifts in the microbial community of natural *Drosophila melanogaster* populations

Marion Margaux Lemoine[1], Thomas Wöhner[2] and Martin Kaltenpoth[1]*

[1]Max Planck Institute for Chemical Ecology, Jena, Germany

[2]Julius Kühn-Institut (JKI), Federal Research Centre for Cultivated Plants, Institute for Breeding Research on Fruit Crops, Dresden, Germany

*Corresponding author: Martin Kaltenpoth, Department of Insect Symbiosis, Max Planck Institute for Chemical Ecology, Jena, Germany, kaltenpoth@ice.mpg.de

Table S1: Primer pairs used for PCR and qPCR essays. Ta: Annealing temperature

	Target	Primer name	Primer sequence		Ta	Reference
PCR	*Drosophila melanogaster*	Droso_S391	fwd	AAATAACAATACAGGACTCATATCC	50°C	Naserzadeh et al.2020 (1)
		Droso_A381	rev	GTAATACGCTTACATACATAAAGGTATA		
qPCR	Eubacteria	EUB338mod	fwd	TCCTACGGGAGGCAGCAG	55°C	Fierer et al. 2005 (2)
		EUB518	rev	ATTACCGCGGCTGCTGG		
	Fungi	FR1	fwd	CGATAACGAACGAGACCT	50°C	Chemidlin Prévost-Bouré et al. 2011 (3)
		FF390	rev	AICCATTCAATCGGTAIT		
	Wolbachia	Wolb_16S	fwd	TTGCTATTAGATGAGCCTATATTAG	55°C	Makepeace et al. 2006 (4)
		Wolb_16S	rev	GTGTGGCTGATCATCCTCT		

Table S2: Result of the indicator species analysis on the bacterial communities of *Drosophila melanogaster* for all sampling sites combined. *ANPR**:Allorhizobium-Neorhizobium-Pararhizobium-Rhizobium,*

ASV number	genus	species	Associated month					specificity coefficient	p-value
			April	May	June	July	August		
113	*Bacillus*	na	x					0.64	0.006
131	*Bacillus*	na	x					0.59	0.010
243	*Orbus*	na	x					0.60	0.003
250	*Glutamicibacter*	na	x					0.53	0.020
254	*Massilia*	na	x					0.53	0.029
262	*Orbus*	na	x					0.42	0.034
266	*Orbus*	na	x					0.43	0.036
345	*Massilia*	*timonae*	x					0.51	0.026
357	*Staphylococcus*	*hominis*	x					0.43	0.034
365	*Staphylococcus*	na	x					0.42	0.026
385	*Rhizorhapis*	na	x					0.48	0.037
388	*Tanticharoenia*	na	x					0.63	0.002
477	*Tepidimonas*	*fonticaldi*	x					0.63	0.005
503	*Bacillus*	na	x					0.62	0.003
634	*Staphylococcus*	na	x					0.43	0.034
706	*Bacillus*	na	x					0.63	0.005
720	*Streptococcus*	na	x					0.39	0.034
722	Kytococcus	na	x					0.64	0.004
736	*Rothia*	na	x					0.44	0.009
771	*ANPR**	na	x					0.41	0.046
805	*Bryocella*	na	x					0.63	0.003
821	*Bacillus*	na	x					0.42	0.039
912	*Pseudoxanthomonas*	*taiwanensis*	x					0.63	0.004
1116	*Enterobacter*	na	x					0.63	0.004
1138	*Citrobacter*	na	x					0.43	0.026
1223	*Afipia*	na	x					0.43	0.033
1241	*Staphylococcus*	na	x					0.39	0.039
1414	*Enterococcus*	na	x					0.58	0.005
1421	*Sphingobium*	na	x					0.43	0.040
1455	*Brachybacterium*	na	x					0.41	0.035
1673	*Dietzia*	na	x					0.41	0.035
1700	*Sphingomonas*	na	x					0.42	0.042
1730	*Abiotrophia*	na	x					0.43	0.043
1973	*Weissella*	na	x					0.63	0.004
1996	*Vibrio*	na	x					0.41	0.026
2135	*Mycobacterium*	na	x					0.42	0.024
2153	*Macrococcus*	na	x					0.43	0.024
2313	*Streptococcus*	na	x					0.44	0.035
2660	*Macrococcus*	na	x					0.42	0.027
7	*Gluconobacter*	na	x	x				0.83	0.001
52	*Acetobacter*	na	x	x				0.62	0.007
53	*Acetobacter*	na	x	x				0.58	0.027
66	*Orbus*	na	x	x				0.54	0.033
67	*Orbus*	na	x	x				0.54	0.023
231	*Glutamicibacter*	na	x	x				0.57	0.015
304	*Escherichia-Shigella*	na	x		x			0.39	0.042
346	*Dysgonomonas*	na	x		x			0.43	0.048
41	*Orbus*	na	x	x	x			0.61	0.042
45	*Gluconobacter*	na	x	x	x			0.59	0.032

47	*Erwinia*	na	x	x	x			0.68	0.020
470	*Erwinia*	na	x	x	x			0.57	0.047
599	*Dolosigranulum*	na		x				0.45	0.025
109	*Tatumella*	na		x				0.47	0.012
107	*Tatumella*	*punctata*		x				0.47	0.012
8	*Gluconobacter*	na	x	x	x	x		0.82	0.003
598	*Erwinia*	na		x	x	x		0.60	0.024
627	*Corynebacterium*	na				x		0.41	0.026
65	*Komagataeibacter*	na				x	x	0.51	0.043
69	*Acetobacter*	na				x	x	0.51	0.031
71	*Acetobacter*	na				x	x	0.46	0.038
178	*Oenococcus*	na				x	x	0.53	0.037
73	*Acetobacter*	na					x	0.58	0.030
82	*Komagataeibacter*	na					x	0.66	0.013
87	*Komagataeibacter*	na					x	0.68	0.010
115	*Komagataeibacter*	na					x	0.62	0.014
117	*Komagataeibacter*	na					x	0.66	0.006
146	*Acetobacter*	na					x	0.44	0.033
153	Komagataeibacter	na					x	0.51	0.022
157	*Komagataeibacter*	na					x	0.46	0.026
169	*Acetobacter*	na					x	0.53	0.032
182	*Oenococcus*	na					x	0.53	0.032
196	*Komagataeibacter*	na					x	0.53	0.036
203	*Acetobacter*	na					x	0.47	0.047
209	*Komagataeibacter*	na					x	0.54	0.029
265	*Acetobacter*	na					x	0.45	0.028
290	*Acetobacter*	na					x	0.41	0.017
494	*Taibaiella*	na					x	0.47	0.016
702	*ANPR**	na					x	0.41	0.018
913	*Leucobacter*	na					x	0.41	0.022
1328	*Flavobacterium*	na					x	0.41	0.016
2055	*Streptomyces*	na					x	0.41	0.029
632	*Sphingomonas*	na	x				x	0.47	0.033
149	*Acetobacter*	*okinawensis*		x		x		0.54	0.022
151	*Acetobacter*	na		x			x	0.51	0.032
46	*Erwinia*	na	x	x	x		x	0.70	0.021
55	*Bacillus*	na	x	x	x		x	0.72	0.010
63	*Bacillus*	na	x	x	x		x	0.72	0.009

Table S3: Result of the indicator species analysis on the bacterial communities of *Drosophila melanogaster* for Dresden samples.

ASV number	genus	species	Associated month					specificity coefficient	p-value
			April	May	June	July	August		
243	*Orbus*	na	x					0.63	0.029
388	*Tanticharoenia*	na	x					0.63	0.039
477	*Tepidimonas*	*fonticaldi*	x					0.63	0.039
503	*Bacillus*	na	x					0.61	0.035
706	*Bacillus*	na	x					0.62	0.042
722	*Kytococcus*	na	x					0.77	0.006
805	*Bryocella*	na	x					0.63	0.033
912	*Pseudoxanthomonas*	*taiwanensis*	x					0.63	0.034
1116	*Enterobacter*	na	x					0.63	0.034
1973	*Weissella*	na	x					0.63	0.034
7	*Gluconobacter*	na	x	x				0.90	0.014
8	*Gluconobacter*	na	x	x				0.90	0.010
268	*Bacillus*	na	x	x				0.69	0.015
113	*Bacillus*	*na*	x	x	x			0.73	0.046
18	*Paenibacillus*	na		x	x			0.73	0.020
74	*Pseudoxanthomonas*	na		x	x			0.73	0.012
164	*Erwinia*	na		x	x			0.64	0.035
598	*Erwinia*	na		x	x			0.70	0.036
1118	*Streptomyces*	na			x			0.63	0.032
58	*Taibaiella*	na					x	0.62	0.049
73	*Acetobacter*	na					x	0.90	0.001
81	*Acetobacter*	*aceti*					x	0.75	0.005
82	*Komagataeibacter*	na					x	0.94	0.001
87	*Komagataeibacter*	na					x	0.95	0.001
115	*Komagataeibacter*	na					x	0.91	0.001
117	*Komagataeibacter*	na					x	0.91	0.001
209	*Komagataeibacter*	na					x	0.68	0.016
401	*Xanthobacter*	na					x	0.71	0.011
494	*Taibaiella*	na					x	0.71	0.012
688	*Aquamicrobium*	*na*					x	0.71	0.013
1328	*Flavobacterium*	na					x	0.71	0.013
28	*Komagataeibacter*	na			x		x	0.79	0.014
66	*Orbus*	na	x				x	0.67	0.030

Table S4: Result of the indicator species analysis on the bacterial communities of *Drosophila melanogaster* for Pirna samples.

ASV number	genus	species	Associated month: May	July	August	specificity coefficient	p-value
41	*Orbus*	na	x			0.98	0.001
42	*Orbus*	na	x			0.98	0.001
66	*Orbus*	na	x			0.98	0.001
67	*Orbus*	na	x			1.00	0.001
79	*Micrococcus*	*luteus*	x			0.80	0.020
90	*Stenotrophomonas*	na	x			0.74	0.030
107	*Tatumella*	*punctata*	x			0.82	0.018
109	*Tatumella*	na	x			0.82	0.018
137	*Corynebacterium*	na	x			0.79	0.018
149	*Acetobacter*	*okinawensis*	x			0.95	0.002
33	*Enterococcus*	na		x		0.82	0.014
69	*Acetobacter*	na		x		0.97	0.002
71	*Acetobacter*	na		x		0.79	0.025
62	*Komagataeibacter*	na		x	x	0.86	0.022
65	*Komagataeibacter*	na		x	x	0.91	0.008
82	*Komagataeibacter*	na		x	x	0.87	0.038
182	*Oenococcus*	na			x	0.80	0.023
9	*Acetobacter*	na	x		x	0.95	0.001
11	*Acetobacter*	na	x		x	0.91	0.007
29	*Acinetobacter*	na	x		x	0.87	0.019
50	*Xylophilus*	na	x		x	0.79	0.039
59	*Undibacterium*	na	x		x	0.87	0.022
94	*Staphylococcus*	na	x		x	0.80	0.037
151	*Acetobacter*	na	x		x	0.85	0.043

Table S5: Result of the indicator species analysis on the bacterial communities of *Drosophila melanogaster* for Meissen samples.

			Associated month					
**ASV number**	**genus**	**species**	May	June	July	August	**specificity coefficient**	**p-value**
52	*Acetobacter*	na	x				0.91	0.001
53	*Acetobacter*	na	x				0.91	0.001
200	*Enhydrobacter*	na	x				0.72	0.022
329	*Bacillus*	*flexus*	x				0.63	0.032
343	*Pseudomonas*	na	x				0.66	0.015
23	*Acetobacter*	na			x		0.77	0.008
26	*Acetobacter*	na			x		0.76	0.009
141	*Acetobacter*	na			x		0.76	0.003
178	*Oenococcus*	na			x		0.78	0.005
182	*Oenococcus*	na			x		0.71	0.015
261	*Ameyamaea*	na			x		0.71	0.008
9	*Acetobacter*	na			x	x	0.81	0.013
11	*Acetobacter*	na			x	x	0.80	0.011
168	*Leuconostoc*	*pseudomesenteroides*			x	x	0.64	0.046
374	*Xanthobacter*	na				x	0.71	0.015
514	*Massilia*	*timonae*				x	0.71	0.016
7	*Gluconobacter*	na	x		x		0.78	0.034
3	*Komagataeibacter*	na	x		x	x	0.88	0.015
4	*Komagataeibacter*	*europaeus*	x		x	x	0.85	0.014
20	*Undibacterium*	na	x	x		x	0.88	0.008
31	*Herbaspirillum*	*frisingense*	x	x		x	0.88	0.026

Table S6: Result of the indicator species analysis on the fungal communities of *Drosophila melanogaster* for all sampling sites combined

			Associated month						
**ASV number**	**genus**	**species**	April	May	June	July	August	**specificity coefficient**	**p-value**
13	*Hanseniaspora*	*mollemarum*	x					0.96	0.001
24	Kregervanrija	*fluxuum*	x					0.92	0.001
25	*Kregervanrija*	*pseudodelftensis*	x					0.78	0.002
31	*Kregervanrija*	*pseudodelftensis*	x					0.86	0.001
33	*Saccharomycopsis*	*oosterbeekiorum*	x					0.96	0.001
49	*Pichia*	*gijzeniarum*	x					0.69	0.002
93	*Zygosaccharomyces*	*microellipsoides*	x					0.70	0.002
113	*Penicillium*	*miczynskii*	x					0.87	0.001
138	*Pichia*	*gijzeniarum*	x					0.45	0.033
153	*Vishniacozyma*	*tephrensis*	x					0.57	0.020
160	*Starmerella*	*stellata*	x					0.41	0.047
161	*Botrytis*	*caroliniana*	x					0.71	0.001
197	*Mycena*	*mirata*	x					0.41	0.045
200	*Krasilnikovozyma*	na	x					0.63	0.003
231	na	na	x					0.72	0.001
293	*Eukaryota**	na	x					0.44	0.009
415	na	na	x					0.63	0.003
501	*Penicillium*	na	x					0.45	0.047
529	na	na	x					0.45	0.047
625	*Cercozoa***	na	x					0.45	0.047
657	*Penicillium*	*bialowiezense*	x					0.45	0.047
15	*Cyberlindnera*	*jadinii*	x	x				0.78	0.022
76	*Pichia*	*nakasei*	x	x				0.52	0.025
183	*Kurtzmaniella*	na	x	x				0.42	0.027
64	*Zygotorulaspora*	*florentina*	x		x			0.63	0.009
4	*Starmerella*	*stellata*	x	x	x			0.71	0.010
6	*Zygosaccharomyces*	*lentus*	x	x	x			0.80	0.008
27	*Pichia*	*nakasei*	x	x	x			0.72	0.005
34	*Penicillium*	na	x	x	x			0.82	0.001
69	*Hanseniaspora*	*osmophila*	x	x	x			0.60	0.025
178	*Saturnispora*	*diversa*		x				0.46	0.034
205	*Hanseniaspora*	*guilliermondii*		x				0.44	0.041
301	*Schwanniomyces*	*occidentalis*		x				0.41	0.022
12	*Starmerella*	*stellata*		x	x	x		0.85	0.001
16	*Starmerella*	*stellata*		x	x	x		0.84	0.001
17	*Saturnispora*	*diversa*		x	x	x		0.67	0.029
48	*Starmerella*	*stellata*			x			0.79	0.001
67	*Kurtzmaniella*	na			x			0.48	0.043
108	*Starmerella*	*stellata*			x			0.57	0.010
130	*Pichia*	*eremophila*			x			0.48	0.017
3	*Hanseniaspora*	*uvarum*		x	x	x	x	0.89	0.020
7	*Pichia*	na		x	x	x	x	0.81	0.016
9	*Pichia*	*eremophila*		x	x	x	x	0.79	0.019
38	*Candida*	*pseudolambica*			x	x		0.55	0.040
44	*Starmerella*	*stellata*			x	x		0.55	0.037
10	*Zygoascus*	*meyerae*			x	x	x	0.76	0.008
50	*Starmera*	*stellimalicola*				x		0.46	0.025
333	*Kurtzmaniella*	*quercitrusa*				x		0.44	0.048
94	*Trigonopsis*	*vinaria*				x	x	0.53	0.031
8	*Saccharomycopsis*	*schoenii*					x	0.84	0.001
20	*Zygosaccharomyces*	*bailii*					x	0.60	0.022

29	*Pichia*	*mandshurica*					x	0.66	0.007
32	*Wickerhamiella*	*kurtzmanii*					x	0.55	0.024
55	*Pichia*	*mandshurica*					x	0.66	0.006
60	*Dipodascopsis*	na					x	0.65	0.011
68	*Penicillium*	*paneum*					x	0.68	0.004
86	*Pichia*	*mandshurica*					x	0.52	0.032
101	*Wickerhamiella*	*kurtzmanii*					x	0.43	0.045
103	*Priceomyces*	*carsonii*					x	0.71	0.002
125	*Saccharomycodes*	*ludwigii*					x	0.46	0.049
150	*Trigonopsis*	*variabilis*					x	0.47	0.028
221	*Wickerhamomyces*	*xylosivorus*					x	0.41	0.024
256	*Trigonopsis*	*variabilis*					x	0.47	0.013
52	*Chytridiomycota****	na	x			x	x	0.70	0.005

Eukaryota: Eukaryota_gen_Incertae_sedis,*
*Cercozoa**: Cercozoa_gen_Incertae_sedis,*
*Chytridiomycota***: Chytridiomycota_gen_Incertae_sedis.*

Table S7: Result of the indicator species analysis on the fungal communities of *Drosophila melanogaster* for Dresden samples.

			Associated month						
**ASV number**	**genus**	**species**	April	May	June	July	August	**specificity coefficient**	**p-value**
31	*Kregervanrija*	*pseudodelftensis*	x					0.84	0.001
33	*Saccharomycopsis*	*oosterbeekiorum*	x					0.96	0.001
161	*Botrytis*	*carolinia*	x					0.77	0.005
200	*Krasilnikovozyma*	na	x					0.63	0.049
231	na	na	x					0.66	0.049
415	na	na	x					0.63	0.049
13	*Hanseniaspora*	*mollemarum*	x	x				1.00	0.001
24	*Kregervanrija*	*fluxuum*	x	x				0.94	0.001
25	*Kregervanrija*	*pseudodelftensis*	x	x				0.85	0.002
27	*Pichia*	*kasei*	x	x				0.74	0.046
34	*Penicillium*	na	x	x				1.00	0.001
49	*Pichia*	*gijzeniarum*	x	x				0.84	0.003
93	*Zygosaccharomyces*	*microellipsoides*	x	x				0.74	0.008
113	*Penicillium*	*miczynskii*	x	x				0.85	0.002
4	*Starmerella*	*stellata*	x	x	x			0.94	0.001
64	*Zygotorulaspora*	*florenti*	x	x	x			0.83	0.002
84	*Pichia*	*kasei*		x				0.67	0.042
12	*Starmerella*	*stellata*			x			0.85	0.001
16	*Starmerella*	*stellata*			x			0.85	0.001
48	*Starmerella*	*stellata*			x			0.94	0.001
61	*Pichia*	*terricola*			x			0.73	0.005
120	*Pichia*	*kudriavzevii*			x			0.63	0.033
223	*Hanseniaspora*	*uvarum*			x			0.63	0.033
7	*Pichia*	na			x	x	x	0.86	0.007
9	*Pichia*	*eremophila*			x	x	x	0.81	0.020
151	*Martiniozyma*	*asiatica*				x		0.64	0.047
10	*Zygoascus*	*meyerae*				x	x	0.91	0.001
94	*Trigonopsis*	*viria*				x	x	0.71	0.035
20	*Zygosaccharomyces*	*bailii*					x	0.80	0.002
32	*Wickerhamiella*	*kurtzmanii*					x	0.99	0.001
42	*Pichia*	*mandshurica*					x	0.76	0.013
43	*Trigonopsis*	*viria*					x	0.66	0.032
46	*Zygosaccharomyces*	*bisporus*					x	0.71	0.013
55	*Pichia*	*mandshurica*					x	0.87	0.002
60	*Dipodascopsis*	na					x	0.81	0.003
68	*Penicillium*	*paneum*					x	0.69	0.015
73	*Wickerhamomyces*	*anomalus*					x	0.64	0.037
101	*Wickerhamiella*	*kurtzmanii*					x	0.77	0.006
103	*Priceomyces*	*carsonii*					x	0.77	0.001
194	*Myxozyma*	*udenii*					x	0.71	0.010
256	*Trigonopsis*	*variabilis*					x	0.71	0.010

Table S8: Result of the indicator species analysis on the fungal communities of *Drosophila melanogaster* for Pirna samples.

ASV number	genus	species	Associated month			specificity coefficient	p-value
			May	July	August		
7	*Pichia*	na	x			0.95	0.006
17	*Saturnispora*	*diversa*	x			0.99	0.001
22	*Pichia*	*eremophila*	x			0.96	0.002
28	*Pichia*	*eremophila*	x			0.90	0.017
116	*Hanseniaspora*	*pseudoguilliermondii*	x			0.94	0.002
178	*Saturnispora*	*diversa*	x			0.91	0.002
205	*Hanseniaspora*	*guilliermondii*	x			0.82	0.016
9	*Pichia*	*eremophila*	x	x		0.93	0.030
12	*Starmerella*	*stellata*	x	x		0.93	0.005
16	*Starmerella*	*stellata*	x	x		0.93	0.004
111	*Candida*	*californica*	x	x		0.82	0.032
30	*Cladosporium*	*herbarum*		x		0.83	0.042
48	*Starmerella*	*stellata*		x		0.77	0.018
52	*Chytridiomycota**	na		x	x	0.96	0.002
8	*Saccharomycopsis*	*schoenii*			x	0.99	0.003
29	*Pichia*	*mandshurica*			x	0.99	0.002
55	*Pichia*	*mandshurica*			x	0.82	0.013
60	*Dipodascopsis*	na			x	0.82	0.013
68	*Penicillium*	*paneum*			x	0.88	0.016
70	*Groenewaldozyma*	*tartarivorans*			x	0.76	0.013
72	*Saccharomycodes*	*ludwigii*			x	0.86	0.009
103	*Priceomyces*	*carsonii*			x	0.91	0.004
125	*Saccharomycodes*	*ludwigii*			x	0.82	0.012
150	*Trigonopsis*	*variabilis*			x	0.82	0.013

Chytridiomycota: Chytridiomycota_gen_Incertae_sedis.*

Table S9: Result of the indicator species analysis on the fungal communities of *Drosophila melanogaster* for Meissen samples.

			**Associated month**					
**ASV number**	**genus**	**species**	May	June	July	August	**specificity coefficient**	**p-value**
4	*Starmerella*	*stellata*	x				0.78	0.018
13	*Hanseniaspora*	*mollemarum*	x				0.84	0.001
53	*Malassezia*	*restricta*	x				0.76	0.034
27	*Pichia*	*nakasei*	x	x			0.83	0.002
17	*Saturnispora*	*diversa*		x			0.80	0.014
69	*Hanseniaspora*	*osmophila*		x			0.71	0.041
34	*Penicillium*	na	x	x	x		0.87	0.008
44	*Starmerella*	*stellata*		x	x		0.77	0.031
48	*Starmerella*	*stellata*		x	x		0.84	0.024
9	*Pichia*	*eremophila*		x	x	x	0.82	0.048
50	*Starmera*	*stellimalicola*			x		0.82	0.005
87	*Groenewaldozyma*	*salmanticensis*			x		0.67	0.023
94	*Trigonopsis*	*vinaria*			x		0.71	0.012
294	*Starmera*	*stellimalicola*			x		0.71	0.016
333	*Kurtzmaniella*	*quercitrusa*			x		0.69	0.020
8	*Saccharomycopsis*	*schoenii*			x	x	0.76	0.034
14	*Saccharomycopsis*	*crataegensis*				x	0.79	0.005
26	*Monilinia*	*polystroma*				x	0.71	0.007
40	*Saccharomyces*	na	x			x	0.69	0.023
45	*Malassezia*	*restricta*	x			x	0.85	0.009

Table S10: Summary statistics of the LMER ANOVA comparing Shannon alpha-diversity indices for the bacterial and fungal communities, as well as bacterial, *Wolbachia*, and fungal titers. The mixed models included the collection site and month as fixed factors and the DNA extraction batch as random factor. LMER: Shannon index or titer ~ site + month + site*month + 1| DNA extraction batch. p-values <0.05 are highlighted in bold. **The DNA extraction batch was included in the model as a random factor. ***The interaction factor was removed from the model since it did not have a significant impact (p>0.05).

Microbial alpha-diversity (Shannon indices)	Fixed factors**	DF	Chi Sq	*P*
Bacterial community**	month	4	9.45	0.051
	site	2	0.61	0.740
Fungal community	month	4	36.87	**<0.001**
	site	2	8.17	**0.017**
	month x site	5	18.11	**0.003**

qPCR target	Fixed factors**	DF	Chi Sq	*P*
Bacteria	month	4	10.93	**0.027**
	site	2	10.16	**0.006**
	month x site	5	13.15	**0.022**
*Wolbachia****	month	4	8.26	0.083
	site	2	0.16	0.924
Fungi	month	4	7.28	0.122
	site	2	2.40	0.301
	month x site	5	13.51	**0.019**

Table S11: Summary statistics of the pairwise comparisons of the Shannon alpha-diversity index of the fungal community as well as for the bacterial and fungal titters across sampling sites and months. The comparisons were performed following the Tukey method and the p-values were adjusted via the Benjamini-Hochberg (BH) method. p-values <0.05 are highlighted in bold.

			Collection month				
			April	May	June	July	August
**Fungal alpha-diversity**	Dresden	April					
		May	0.746				
		June	**0.009**	**0.003**			
		July	0.575	0.805	**<0.001**		
		August	0.446	0.643	**<0.001**	0.768	
	Meissen	April					
		May					
		June		**0.004**			
		July		**0.045**	0.643		
		August		0.384	0.086	0.384	
	Pirna	April					
		May					
		June					
		July		**0.003**			
		August		0.794		**0.005**	
**Bacterial titer**	Dresden	April					
		May	0.902				
		June	0.771	0.902			
		July	0.947	0.902	0.771		
		August	0.961	0.902	0.771	0.961	
	Meissen	April					
		May					
		June		**0.002**			
		July		0.771	0.063		
		August		0.841	**0.039**	0.944	
	Pirna	April					
		May					
		June					
		July		0.902			
		August		0.944		0.902	
**Fungal titer**	Dresden	April					
		May	0.987				
		June	0.857	0.778			
		July	0.982	0.987	0.778		
		August	0.768	0.718	0.987	0.712	
	Meissen	April					
		May					
		June		0.222			
		July		0.453	**0.007**		
		August		0.768	0.718	0.169	
	Pirna	April					
		May					
		June					
		July		0.982			
		August		0.847		0.791	

Table S12: PERMANOVA results of the comparison of Bray-Curtis distances of the bacterial and fungal communities across sampling sites and months and their interaction. p-values <0.05 are highlighted in bold.

Microbial beta-diversity	Site	Factor	DF	R^2	*P*
Bacteria	all sites	month	4	0.07	**0.001**
		site	2	0.04	**0.001**
		month x site	5	0.08	**0.001**
		DNA extraction batch	5	0.06	0.061
		residuals	69	0.74	
	Dresden	month	4	0.16	**0.003**
		DNA extraction batch	5	0.14	0.761
		residuals	24	0.70	
	Meissen	month	4	0.12	**0.003**
		DNA extraction batch	5	0.18	**0.006**
		residuals	24	0.70	
	Pirna	month	4	0.22	**0.001**
		DNA extraction batch	5	0.25	0.760
		residuals	24	0.53	
Fungi	all sites	month	4	0.20	**0.001**
		site	2	0.10	**0.001**
		month x site	5	0.12	**0.001**
		DNA extraction batch	5	0.04	0.253
		residuals	69	0.54	
	Dresden	month	4	0.41	**0.001**
		DNA extraction batch	5	0.09	0.636
		residuals	24	0.50	
	Meissen	month	4	0.23	**0.001**
		DNA extraction batch	5	0.14	0.294
		residuals	24	0.63	
	Pirna	month	4	0.42	**0.001**
		DNA extraction batch	5	0.20	0.449
		residuals	24	0.38	

Table S13: Summary of the fungal indicator species for all analysis (all sites combined and all sites individually), at the strain level. Each ASV identified as indicator species in at least one of the analyses is grouped according to its genus and the species name is showed each time it is associated with one or several months.

	April		May		June		July		August	
**genus**	species	ASV	species	ASV	species	ASV	species	ASV	species	ASV
Botrytis	*B.caro-liniana*	161								
*Eukaryota**	*na*	293								
*Cercozoa***	*na*	625								
Krasilnikovozyma	*K.fibulata*●	200								
Mycena	*M.mirata*	197								
na	*na*	415								
Vishniacozyma	*V.tephren-sis*	153								
Cyberlindnera	*C.jadinii*	15	*C.jadinii*	15						
Kregervanrija	*K.fluxuum*	24	*K.fluxuum*	24						
	K.pseudo-delftensis	25 31	*K.pseudodelf tensis*	25						
Schwanniomyces			*S.occidentali s*	301						
Zygotorulaspora	*Z.florentina*	64	*Z.florentina*	64	*Z.florentina*	64				
Kurtzmaniella	*K.fragi*●	183	*K.fragi*●	183						
					na	67				
							K.querci-trusa	33 3		
Hanseniaspora	***H.molle-marum***	**13**	***H.molle-marum***	**13**						
	H.osmophil a	69	*H.osmophila*	69	*H.osmophila*	69				
			H.pseudo-guilliermondii	116						
			H.guillier-mondii	205						
			H.uvarum	3	*H.uvarum*	3223	*H.uvarum*	3	*H.uvarum*	3
Starmerella	***S.stellata***	**4** 160	***S.stellata***	**4 12 16**	***S.stellata***	**4 12 16** 44 **48** 108	***S.stellata***	**12 16** 44 **48**		
Candida			*C.californica*	111			*C.califor-nica*	111		
					C.pseudo-lambica	38	*C.pseudo-lambica*	38		
Saturnispora			***S.diversa***	**17,** 178	***S.diversa***	**17**	***S.diversa***	**17**		
Penicillium	*P. raistrickii*●	501								
	P.bialo-wiezense	657								
	P.miczynski i	113	*P.miczynskii*	113						
	***P.solitum*●**	**34**	***P.solitum*●**	**34**	***P.solitum*●**	**34**	***P.solitum*●**	**34**		
									P.paneum	**68**
Zygosaccharo-myces	*Z.micro-ellipsoides*	93	*Z.micro-ellipsoides*	93						

	Z.lentus	6	*Z.lentus*	6	*Z.lentus*	6				
									Z.bailii	20
									Z. bisporus	46
Pichia	*P.gijze-niarum*	49 138	*P.gijze-niarum*	49						
	P.nakasei	**27** 76	***P.nakasei***	**27** 76	***P.nakasei***	**27**				
			P. eremophila	**9** 22 28	***P. eremophila***	**9** 130	***P.eremophila***	**9**	***P.eremophila***	**9**
			P.kluyveri●	**7**	***P.kluyveri●***	**7**	***P.kluyveri●***	**7**	***P.kluyveri●***	**7**
					P.terricola	61				
					P.kudriavezii	120				
									P.mand-shurica	29 **59** 86
Saccharo-mycopsis	*S.ooster-beekiorum*	33								
							S.schoenii	**8**	***S.schoenii***	**8**
									S.cratae-gensis	14
Zygoascus					*Z.meyerae*	10	*Z.meyerae*	10	*Z.meyerae*	10
Cladosporium							*C.herbarum*	30		
Martiniozyma							*M.asiatica*	151		
Starmera							*S.stellimalicola*	50, 294		
Groenewaldozyma							*G.salmanticensis*	87	*G.tartarivorans*	70
Trigonopsis							***T.vinaria***	**94**	***T.vinaria***	43, **94**
									T.variabilis	150, 256
Dipodascopsis									***D.uninucleata●***	**60**
Wickerhamomyces									*W.xylosivorus*	221
									W.anomalus	73
Monilinia									*M.polystroma*	26
Myxozyma									*M.udenii*	194
Priceomyces									***P.carsonii***	**103**
Saccharomycodes									*S.ludwigii*	72, 125
Wickerhamiella									***W.kurtzmanii***	32, **101**
Malassezia	*M.restricta*	45	*M.restricta*	53					*M.restricta*	45
*Chytridiomycota****	*na*	52					*na*	52	*na*	52
Saccharomyces	*na*	40							*na*	40

Eukaryota: Eukaryota_gen_Incertae_sedis,*
*Cercozoa**:Cercozoa_gen_Incertae_sedis*
*Chytridiomycota***: Chytridiomycota_gen_Incertae_sedis.*
● : ASV identified at the species level via the BLAST tool from NCBI.

Table S14: Procrustes analysis results for the correlation between bacterial and fungal communities for all sites combined and each site individually.

Site	Dissimilarity	Correlation	variance accounted	*P*
all sites	0.91	0.29	8.41	**0.001**
Dresden	0.86	0.38	14.20	**0.018**
Meissen	0.93	0.26	6.63	0.206
Pirna	0.71	0.53	28.54	**0.012**

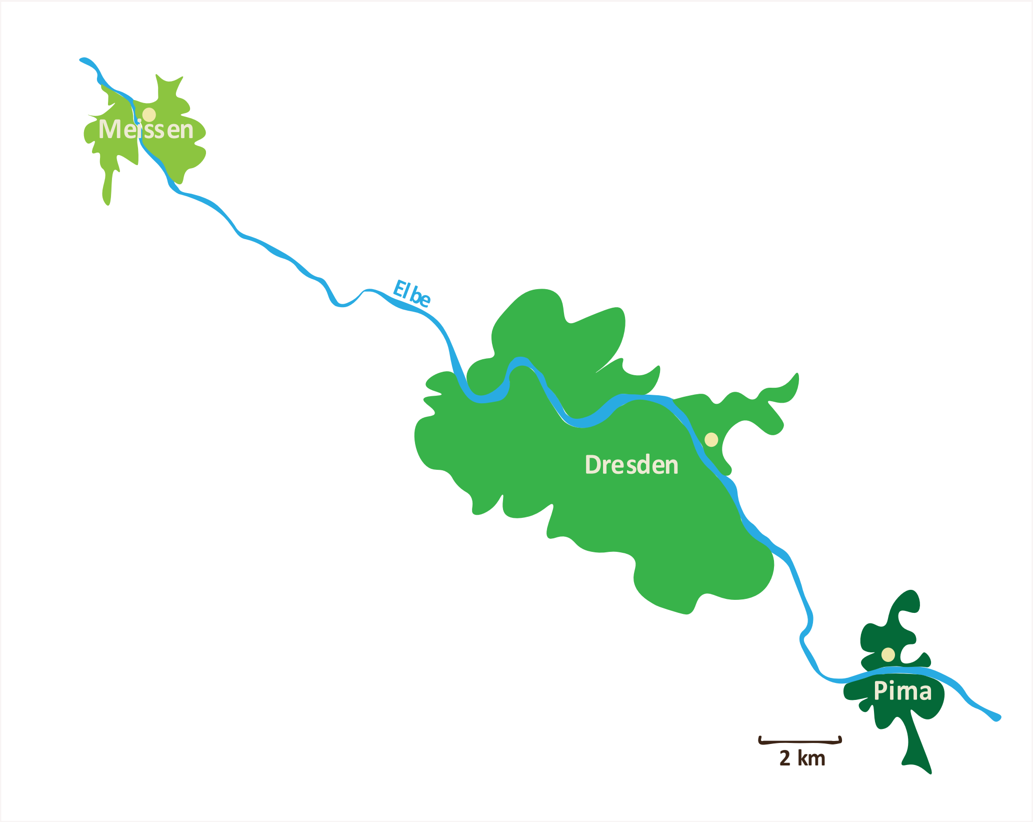

Figure S1: Schematic map of the different sampling sites in Germany where *Drosophila melanogaster* specimens were collected (yellow dots).

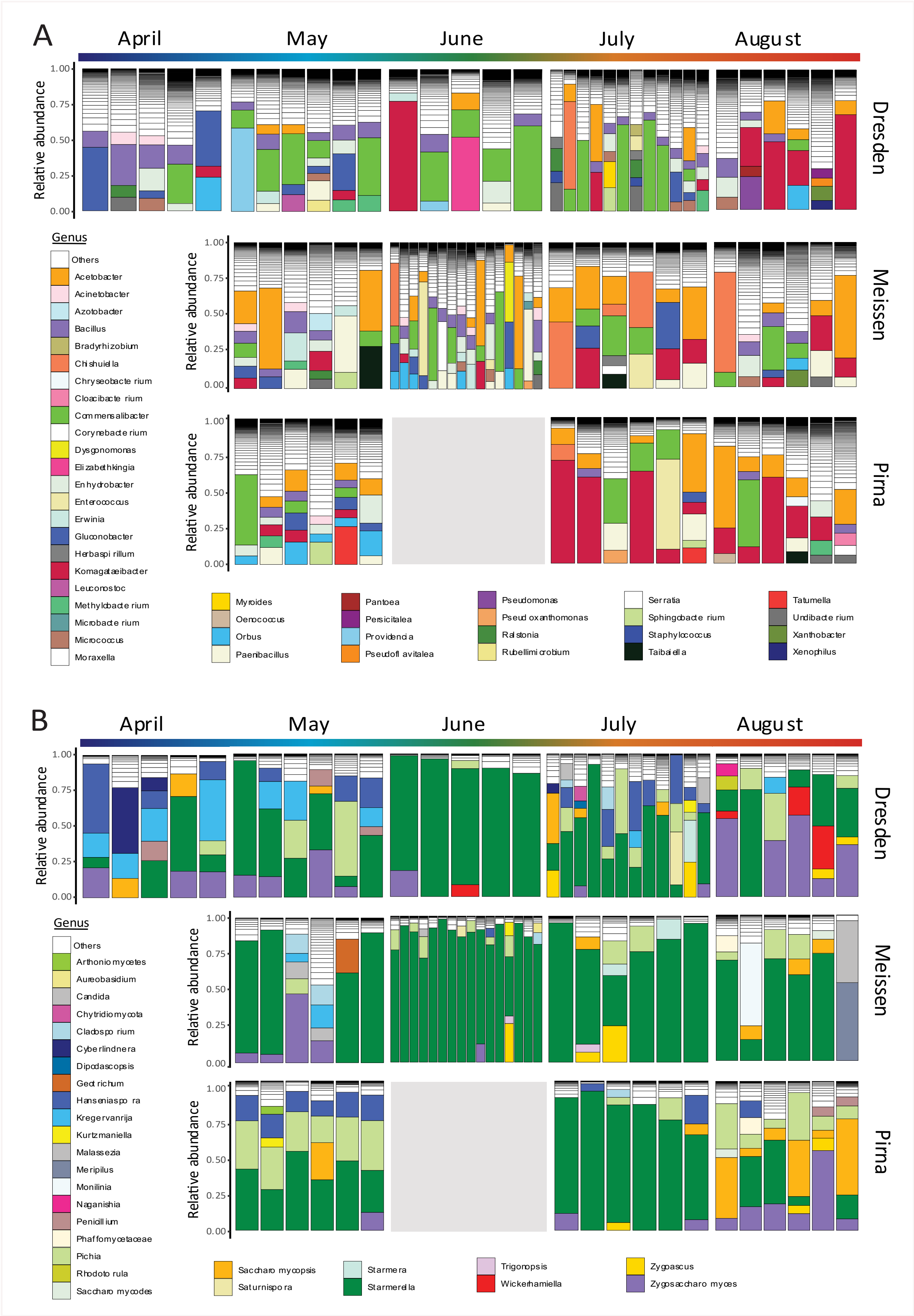
A
April
May
June
July
August
Dresden
Meissen
Pirna
Relative abundance
1.00
0.75
0.50
0.25
0.00
Genus
Others
Acetobacter
Acinetobacter
Azotobacter
Bacillus
Bradyrhizobium
Chishuiella
Chryseobacterium
Cloacibacterium
Commensalibacter
Corynebacterium
Dysgonomonas
Elizabethkingia
Enhydrobacter
Enterococcus
Erwinia
Gluconobacter
Herbaspirillum
Komagataeibacter
Leuconostoc
Methylobacterium
Microbacterium
Micrococcus
Moraxella
Myroides
Oenococcus
Orbus
Paenibacillus
Pantoea
Persicitalea
Providencia
Pseudoflavitalea
Pseudomonas
Pseudoxanthomonas
Ralstonia
Rubellimicrobium
Serratia
Sphingobacterium
Staphylococcus
Taibaiella
Tatumella
Undibacterium
Xanthobacter
Xenophilus
B
April
May
June
July
August
Dresden
Meissen
Pirna
Relative abundance
1.00
0.75
0.50
0.25
0.00
Genus
Others
Arthoniomycetes
Aureobasidium
Candida
Chytridiomycota
Cladosporium
Cyberlindnera
Dipodascopsis
Geotrichum
Hanseniaspora
Kregervanrija
Kurtzmaniella
Malassezia
Meripilus
Monilinia
Naganishia
Penicillium
Phaffomycetaceae
Pichia
Rhodotorula
Saccharomycodes
Saccharomycopsis
Saturnispora
Starmera
Starmerella
Trigonopsis
Wickerhamiella
Zygoascus
Zygosaccharomyces

Figure S2: Bacterial (A) and fungal (B) community of single *Drosophila melanogaster* individuals at the genus level and including the rare ASVs that were omitted in Figure 1 and 4. Coloured sections of each bar show bacterial or fungal genera with a relative abundance above 5% in each sample. The rest of the ASVs, agglomerated in rarer orders, are compiled in the “Others” category.

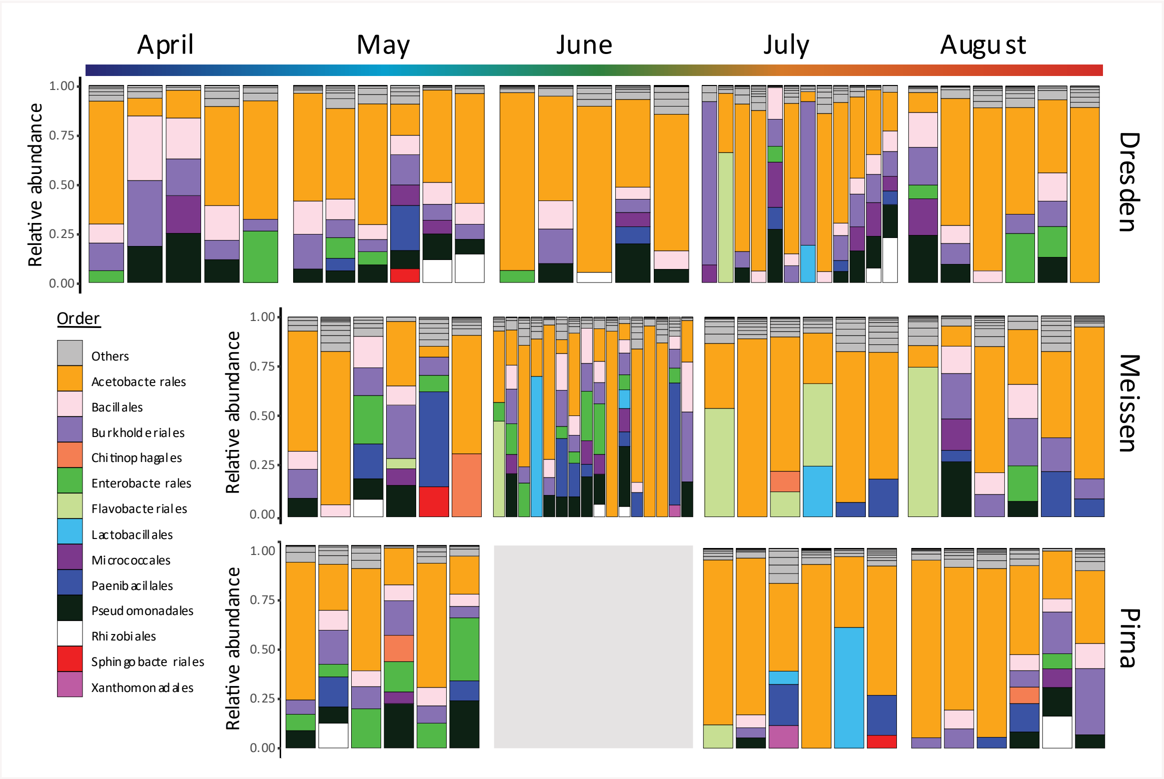

Figure S3: Bacterial community of single *Drosophila melanogaster* individuals at the order level. Coloured sections of each bar show bacterial orders with a relative abundance above 5% in each sample. The rest of the ASVs, agglomerated in rarer orders, are compiled in the “Others”.

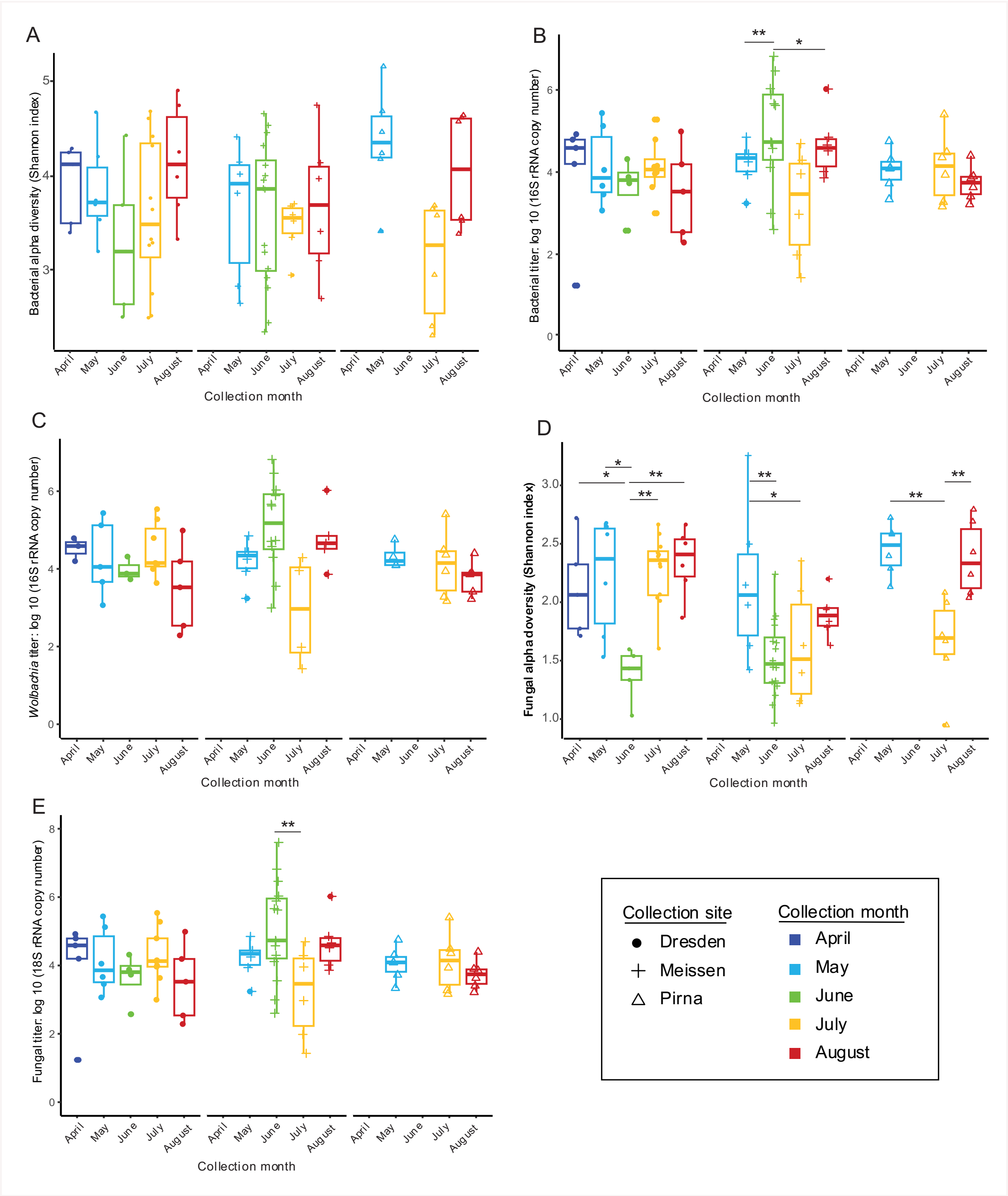

Figure S4: Shannon alpha-diversity indices for the bacterial and fungal communities (A and D) and total bacterial, *Wolbachia* and fungal titers (B, C and E) from flies across all collection sites and time points. Asterisks indicate significant differences between groups (LMME and Tukey post-hoc test), *: p-value<0.05, **p-value<0.01.

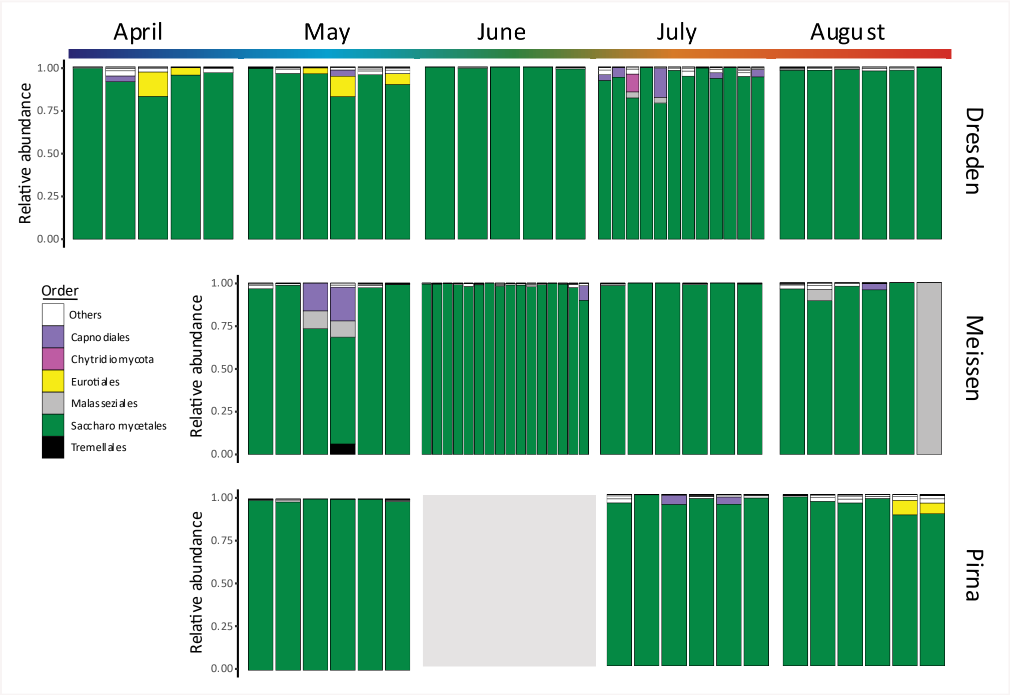

Figure S5: Fungal community profiles of single *Drosophila melanogaster* individuals at the order level. Coloured sections of each bar show fungal orders with a relative abundance above 5% in each sample. The rest of the ASVs, agglomerated in rarer orders, are compiled in the “Others” category.

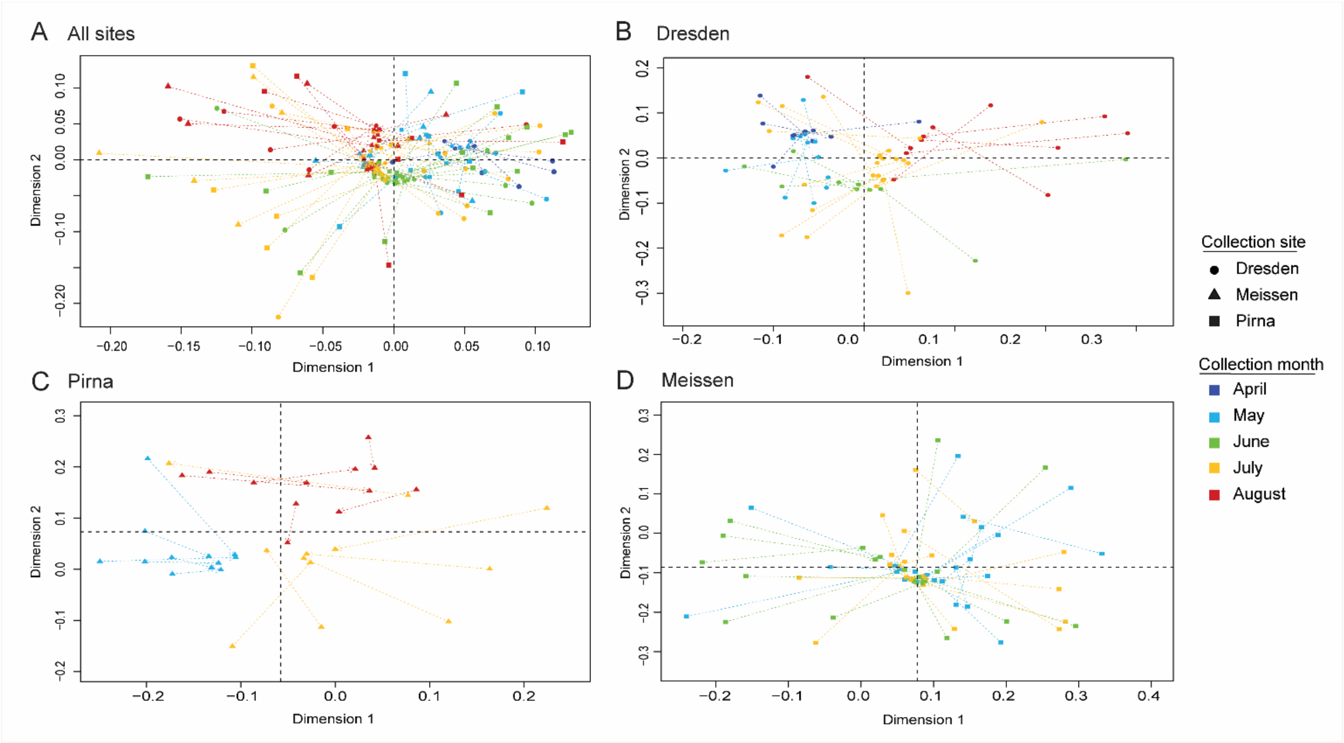

Figure S6: Procrustes analyses for correlations between bacterial and fungal communities for all sites combined (A) and for each individual site (B to D). Each point, representing the bacterial or fungal community of a single fly, is colored according to the sampling month, and shaped according to the sampling site. The dashed lines connect the bacterial community (central point) and fungal community (external point) of each fly. The lines length indicates the strength of the correlation, shorter arrows show stronger correlation among connected points.

References

1. Naserzadeh Y, Pakina EN, Nafchi AM, Gadzhikurbanov AS. Specific Identification Method based on PCR for Drosophila melanogaster. RUDN: AA. 2020;15(2):134-41.
2. Fierer N, Jackson JA, Vilgalys R, Jackson RB. Assessment of soil microbial community structure by use of taxon-specific quantitative PCR assays. Appl Environ Microbiol. 2005 Jul;71(7):4117-20.
3. Chemidlin Prévost-Bouré N, Christen R, Dequiedt S, Mougel C, Lelièvre M, Jolivet C, Shahbazkia HR, Guillou L, Arrouays D, Ranjard L. Validation and application of a PCR primer set to quantify fungal communities in the soil environment by real-time quantitative PCR. PLoS One. 2011;6(9):e24166.
4. Makepeace BL, Rodgers L, Trees AJ. Rate of Elimination of Wolbachia pipientis by Doxycycline In Vitro Increases following Drug Withdrawal. Antimicrob Agents Chemother. 2006; 50.3: 922-927.
